# Micronutrient-Fortified Rice Can Increase Hookworm Infection Risk: A Cluster Randomized Trial

**DOI:** 10.1371/journal.pone.0145351

**Published:** 2016-01-06

**Authors:** Brechje de Gier, Maiza Campos Ponce, Marlene Perignon, Marion Fiorentino, Kuong Khov, Chhoun Chamnan, Michiel R. de Boer, Megan E. Parker, Kurt Burja, Marjoleine A. Dijkhuizen, Jacques Berger, Katja Polman, Frank T. Wieringa

**Affiliations:** 1 Section Health and Life Sciences, Athena Institute, Faculty of Earth and Life Sciences, VU University Amsterdam, Amsterdam, The Netherlands; 2 Section Infectious Diseases, department of Health Sciences, Faculty of Earth and Life Sciences, VU University Amsterdam, Amsterdam, The Netherlands; 3 UMR-204, NutriPass IRD-UM-SupAgro, Institut de Recherche pour le Développement, Montpellier, France; 4 Department of Fisheries Post-Harvest Technologies and Quality control, Fisheries Administration, Phnom Penh, Cambodia; 5 Section Methodology and Applied Biostatistics, Department of Health Sciences, Faculty of Earth and Life Sciences, VU University Amsterdam, Amsterdam, The Netherlands; 6 PATH, Seattle, Washington, United States of America; 7 United Nations World Food Programme, Phnom Penh, Kingdom of Cambodia; 8 Department of Nutrition, Exercise and Sports, Copenhagen University, Copenhagen, Denmark; 9 Department of Biomedical Sciences, Institute for Tropical Medicine, Antwerp, Belgium; The George Washington University School of Medicine and Health Sciences, UNITED STATES

## Abstract

**Background:**

Fortification of staple foods is considered an effective and safe strategy to combat micronutrient deficiencies, thereby improving health. While improving micronutrient status might be expected to have positive effects on immunity, some studies have reported increases in infections or inflammation after iron supplementation.

**Objective:**

To study effects of micronutrient-fortified rice on hookworm infection in Cambodian schoolchildren.

**Methods:**

A double-blinded, cluster-randomized trial was conducted in 16 Cambodian primary schools partaking in the World Food Program school meal program. Three types of multi-micronutrient fortified rice were tested against placebo rice within the school meal program: UltraRice_original, UltraRice_improved and NutriRice. Four schools were randomly assigned to each study group (placebo n = 492, UltraRice_original n = 479, UltraRice_improved n = 500, NutriRice n = 506). Intestinal parasite infection was measured in fecal samples by Kato-Katz method at baseline and after three and seven months. In a subgroup (N = 330), fecal calprotectin was measured by ELISA as a marker for intestinal inflammation.

**Results:**

Baseline prevalence of hookworm infection was 18.6%, but differed considerably among schools (range 0%- 48.1%).Micronutrient-fortified rice significantly increased risk of new hookworm infection. This effect was modified by baseline hookworm prevalence at the school; hookworm infection risk was increased by all three types of fortified rice in schools where baseline prevalence was high (>15%), and only by UltraRice_original in schools with low baseline prevalence. Neither hookworm infection nor fortified rice was related to fecal calprotectin.

**Conclusions:**

Consumption of rice fortified with micronutrients can increase hookworm prevalence, especially in environments with high infection pressure. When considering fortification of staple foods, a careful risk-benefit analysis is warranted, taking into account severity of micronutrient deficiencies and local prevalence of parasitic infections.

**Trial Registration:**

ClinicalTrials.gov NCT01706419

## Introduction

Micronutrient deficiencies form a large public health problem worldwide, especially in tropical regions[1]. Children are particularly vulnerable, due to their specific nutritional needs for growth and development. Approximately 50% of all child mortality has been attributed to malnutrition, including deficiencies of iron, vitamin A and zinc[2, 3].Aside from mortality, micronutrient deficiencies affect growth and cognitive development[4, 5].Micronutrient deficiencies are frequently combated by micronutrient supplementation or fortification of staple foods [1]. Indeed, the Copenhagen Consensus ranks food fortification as one of the most cost-effective tools to combat malnutrition[6].

The world regions where micronutrient deficiencies are the most common are also often plagued by high prevalence of helminth infections. Associations between micronutrients and helminth infections have been reported, although many questions remain unanswered[7]. Micronutrient deficiencies can increase susceptibility to infection, but infections can also alter the intestinal mucosa, leading to reduced absorption of nutrients. This phenomenon is being increasingly recognized as environmental enteropathy[8]. On the other hand, micronutrient fortification might even increase infection risk or persistence. This phenomenon has been described for iron supplementation and several pathogens[9]. The debate surrounding this conundrum has been fueled by a trial in Pemba, Tanzania, in which mortality for malaria and other infections was higher in children who were given iron and folate supplements[10]. Since then, systematic reviews have been performed but have not found a significantly increased infection risk after iron or multi-micronutrient supplementation[7, 11].

Aside from infections, the intestinal environment might be altered in other ways by micronutrient supplementation. In 2010, Zimmermann et al found increases in intestinal inflammation and enterobacteria after iron supplementation[12]. These findings raise questions about the effects of micronutrient supplementation or fortification on the intestinal environment and immunity.

Despite considerable improvements in health and nutrition since the 1990’s, Cambodian children are at high risk of stunting, wasting and micronutrient deficiencies[13–15]. A 2010 national survey found a prevalence of stunting in children <5 of almost 40%[15]. The prevalence of helminths, mainly of hookworm, is also considerable in Cambodia[16]. The FORISCA UltraRice+NutriRice project was a large scale cluster-randomized, double-blinded, placebo-controlled trial, conducted in 16 primary schools, all situated in one province in Cambodia. The project aimed to quantify the impact of multi-micronutrient fortified rice, which was distributed through the World Food Program (WFP) school meal program as a single meal per day, on micronutrient status, health and cognition of Cambodian schoolchildren. As uncertainly exists on the most optimal combination of micronutrients to be added to rice, 3 different types of fortified rice, produced using different methods and with different micronutrient composition, were studied. Secondary outcomes included helminth infection, anthropometry and intestinal inflammation. Here we report on effects of the introduction of fortified rice on hookworm infection and local intestinal inflammation.

## Methods

### Study design and population

In a double-blinded, cluster-randomized, placebo-controlled trial, three different types of multi-micronutrient fortified rice were introduced through the World Food Program (WFP) School Meal program in Cambodia. The clusters were 16 primary schools in rural Kampong Speu province, of which four were randomly selected for each study group. Schools were eligible if they participated in the WFP school meal program and all children were served breakfast daily. In total 18 schools were eligible, two were excluded because of the number of school children (one school had double the number of school children (N>1200) as the other schools, and one school had <100 school children, whereas for biochemical determination of micronutrient status a minimum of 125 school children was required per school). A cluster-randomization was chosen because the schools had one kitchen each, and separate preparations of school meals were not feasible. The study was powered for its primary outcomes (micronutrient status), which are not reported here. The trial took place from November 2012 to June 2013. The clusters were 16 primary schools in rural Kampong Speu province, of which four were randomly selected for each study group. Calprotectin was measured in a subsample, from two schools from the placebo, UltraRice_original and UltraRice_improved study groups due to financial restraints. Written informed consent of at least one parent was obtained prior to the study. Ethical approval was obtained from the Cambodian Ministry of Health, Education and Planning and the Ethical Review board of PATH, USA. This study population is further described by Perignon et al[17].

### Intervention

The 3 types of fortified rice differed in micronutrient compositions (Table 1), as well as production procedures. NutriRice was produced by hot extrusion by Buhler Food, Wuxi, China. Both types of UltraRice were custom-made for the project, with UltraRice_original produced using cold extrusion techniques by Maple Grove Gluten-free Foods, Ltd, California, USA and UltraRice_improved by the Food Technology department of Kansas State University, USA. Children received one type of fortified rice or placebo (unfortified white rice) six days per week for six months. Aside from rice, the school meals consisted of canned fish, vitamin A+D fortified vegetable oil, yellow split peas and iodized salt. After baseline data collection, all children received a single dose of 500 mg mebendazole.

**Table 1:** Micronutrient composition of the three types of fortified rice. N.I. = not included in premix

Micronutrient	Target value(mg)	UltraRice _original(mg)	UltraRice _improved(mg)	NutriRice (mg)
**Vitamin A (retinol)**	0.3	N.I.	0.64	0.29
**Iron**	7.26	10.67	7.55	7.46
**Zinc**	3.5	3.0	2.0	3.7
**Vitamin B1 (Thiamin)**	0.6	1.1	1.4	0.7
**Vitamin B3 (NE)**	8	N.I.	12	8
**Vitamin B6**	0.65	N.I.	N.I.	0.92
**Folate (DFE)**	0.2	0.2	0.3	0.1
**Vitamin B12**	0.001	N.I.	0.004	0.001

### Measurements

The primary outcome for the current paper is hookworm infection, which was the main intestinal parasite found in this population. Stool samples were collected by providing school children with a sample collection container and requesting them to return the next day to school with a fresh stool sample. Sample containers were collected in the morning, stored in a coolbox with blue ice (4°C) and brought to the National Center for Parasitology, Entomology and Malaria control (CNM) in Phnom Penh in the afternoon. Samples were analyzed by Kato-Katz technique at baseline (before treatment), three months and seven months (end of the intervention) to determine helminth infection[18]. Parasite diagnosis was performed by the National Center for Parasitology, Entomology and Malaria control (CNM), Phnom Penh, Cambodia, and recorded as eggs per gram of feces. No distinction was made between hookworm species. For a subgroup of 330 children, at baseline and after seven months stool samples were frozen (-20°C) and sent to the Institute for Tropical Medicine in Antwerp, Belgium where our secondary outcome calprotectin was measured by ELISA (Calpro AS, Norway), according to the manufacturer’s instructions, with 10% of these samples measured in duplicate for quality control. Due to funding restraints, fecal calprotectin was measured only in a subsample of stool samples collected after seven months from UltraRice_original, UltraRice_improved and placebo groups. Sex and age of the children were obtained by interviews and verified by school records and birth certificates. All measurements were at the individual level.

### Randomization and masking

Three different randomizations, combining different schools to one intervention, were separately generated based on a list number of children per school by iteration to fit the predefined criteria of group size (within 10% of the mean). A researcher not involved in the field work (MAD) blindly picked one of the three randomizations, and allocated each group of schools to an intervention arm. To further assure blinding, each intervention arm of 4 schools was split into two groups of two schools, each given a letter code (A–H). The entire research team and all participants and caregivers were blinded to the allocation. The code was only known to one person with WFP, responsible to allocate the correct type of rice to the right school. The rice packaging was coded with the letter allocated during the randomization (A–H) and did not contain the name of the rice type.

### Statistical analysis

Analyses were done using SPSS software version 22 (IBM, NY, USA). Crude percentage points differences in hookworm prevalence (with corresponding confidence intervals for proportions) between the intervention and placebo groups were calculated. Effects of the interventions on hookworm infection risk were analyzed using generalized linear mixed models. A logistic mixed model with hookworm infection as the binary outcome at three and seven months and random intercepts for school and child ID was used to account for repeated measurements within subjects and clustering of subjects within schools. Covariates were sex, age in quartiles and baseline hookworm prevalence at the school. In the analysis we focused on the effect of the intervention on hookworm infection in children who were uninfected at baseline, in order to estimate new infection rate. The fecal calprotectin data were skewed, which could not be corrected by logarithmic transformation. Therefore, fecal calprotectin was analyzed using mixed model analysis with high fecal calprotectin (>50 mg/kg) at seven months as binary outcome and a random intercept for school clustering. Covariates were sex, age in quartiles and high fecal calprotectin at baseline (binary) [19]. Effect modification was examined by introducing interaction terms into the model, when these showed significant effects (p <0.05), we stratified the analysis.

## Results

**Fig 1:**
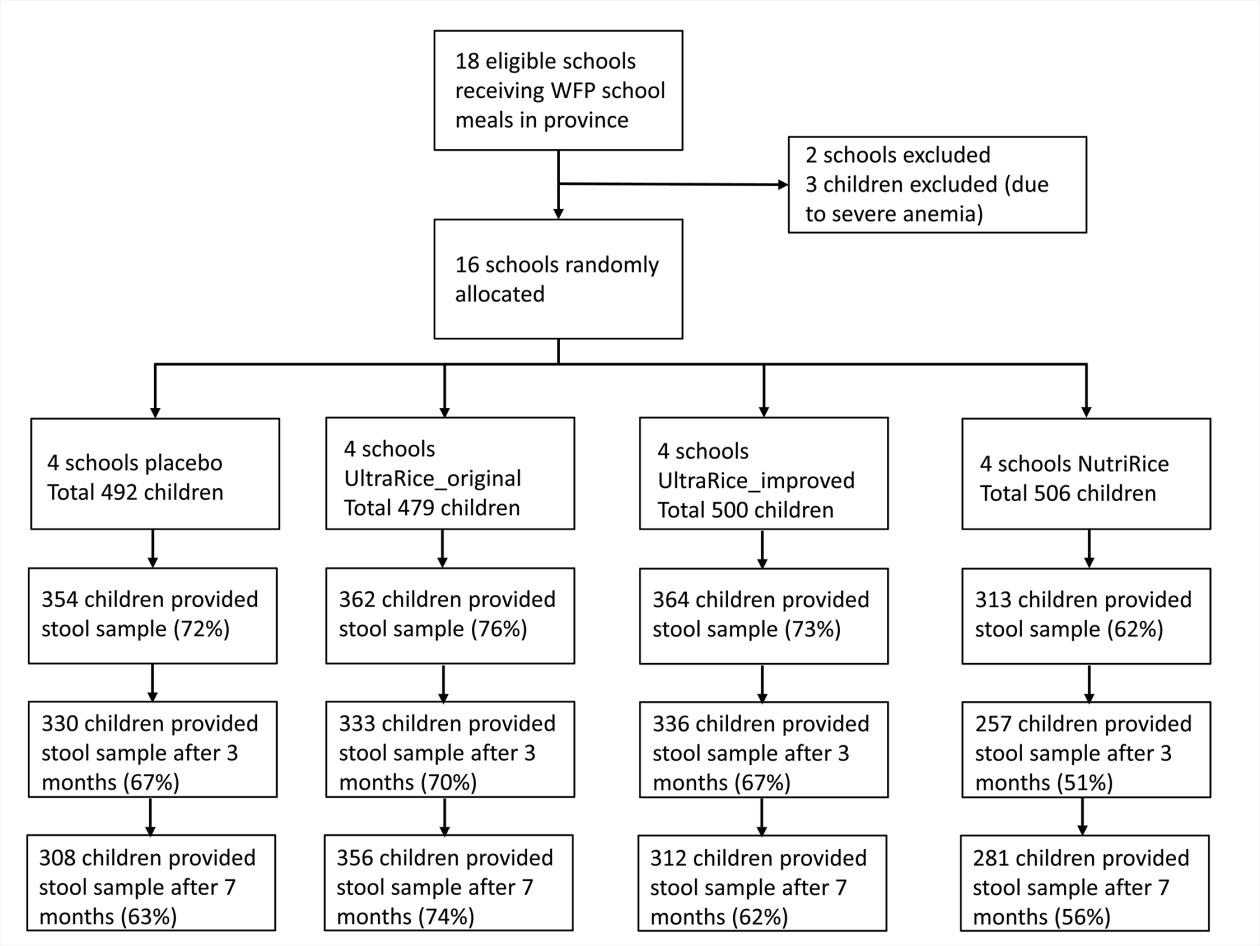
Flow chart of the study.

Rice kernels were analyzed for micronutrient composition by Silliker (Markham, Ontario, Canada) (for both UltraRice kernels) and Buhler (NutriRice kernels; Table 1). A flow chart of all study participants is shown in Fig 1. At baseline, 3 children were excluded due to severe anemia (and received multiple micronutrient supplements for 2 months). Stool samples were obtained from 1393 children at baseline (70.5%, Table 2). After three and seven months, fecal samples were obtained from 1256 (63.5%) and 1257 (63.6%) children, respectively.

**Table 2:** Population characteristics at baseline.

	Placebo	UltraRice_original	UltraRice_improved	Nutririce
**N (male %)**	492 (49.9%)	479 (49.6%)	500 (49.8%)	506 (50.8%)
**Age (mean ± SD)**	9.58 ± 2.27	9.61 ± 2.19	9.64 ± 2.22	9.71 ± 2.42
**Hookworm infection n/N (%)**	74/354 (20.9%)	82/362 (22.7%)	67/364 (18.4%)	36/316 (11.5%)
**Range of baseline hookworm prevalence at schools**	8.0–37.6%	0–48.1%	5.1–33.3%	4.8–26.9%
**Hookworm infection intensity n**				
**-Light**	70	81	65	36
**-Moderate**	4	1	2	0
**-Heavy**	0	0	0	0
**Calprotectin N**	124	117	123	Na
**Calprotectin median (IQR)**	22.7 (21.2 to 26.3)	22.7 (20.2 to 26.9)	21.7 (20.3 to 26.3)	Na
**Calprotectin high n (>50mg/kg)**	13 (10.5%)	7 (6%)	13 (10.6%)	Na

**Fig 2:**
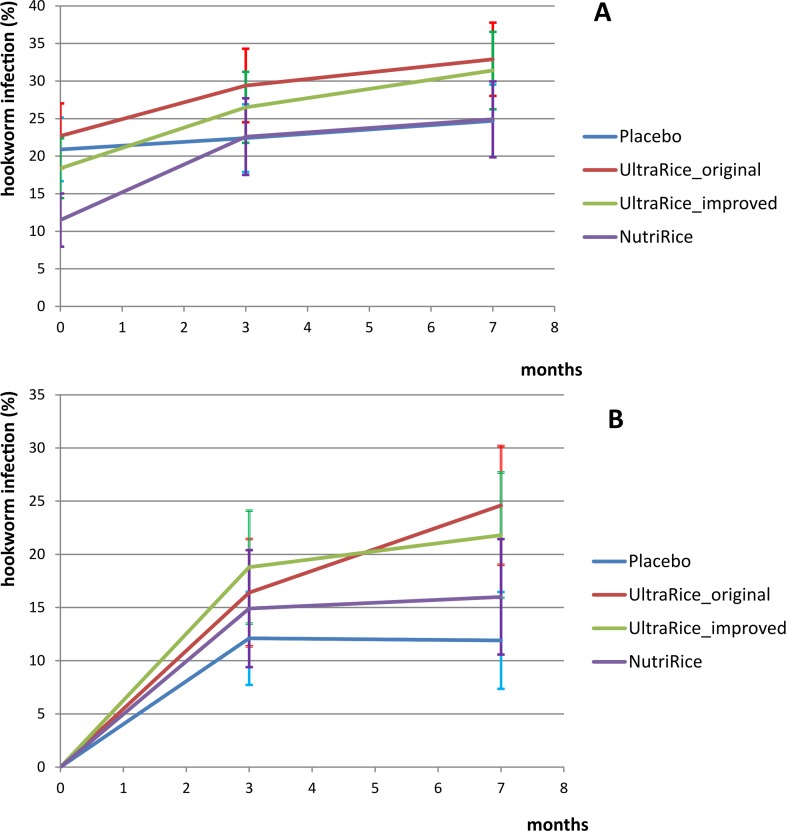
Effect of micronutrient-fortified rice consumption or placebo on hookworm infection. Hookworm infection prevalence in all children (A, N = 1393) and baseline uninfected children (B, N = 1134) per study group, during the course of the intervention, with error bars depicting crude 95% confidence intervals for the proportion infected.

Overall prevalence of hookworm was 18.6% (baseline), 25.4% (three months) and 28.7% (seven months). Hookworm infections were mostly of light intensity (<2000 eggs per gram feces) and no heavy infections (>4000 egg per gram) were found. Baseline prevalence of hookworm in the 16 schools ranged from 0% to 48.1%. Two thirds of the infected children at baseline were also infected after three months. We cannot ascertain whether these are all re-infections or if the anthelminthic treatment provided after baseline had been ineffective, as no stool samples were obtained shortly after treatment. Therefore, to determine whether hookworm infection rate was increased after the introduction of fortified rice, we focused our further analysis on the children who were uninfected at baseline. Fig 2 shows the prevalence of hookworm infections over the course of the study in all included children (A) and baseline uninfected children (B) per intervention group. S1 Fig shows the prevalence of hookworm infection at baseline, after 3 and 7 months for each school, by study group.

New hookworm infection rates were significantly higher in schools receiving any type of fortified rice compared to placebo. The increase in hookworm infection was significant for both types of UltraRice (Table 3, Fig 2B). Baseline prevalence of hookworm at the school level was a significant modifier of the effects of fortified rice on hookworm infection risk. Therefore we stratified for baseline hookworm prevalence (below and above the median of 15%, Table 4). In schools with baseline hookworm prevalence below 15%, only UltraRice_original increased infection risk. In schools with baseline prevalence above 15%, all three types of fortified rice significantly increased the risk of infection compared to placebo.

**Table 3:** Longitudinal effects of micronutrient fortified rice on hookworm infection risk of baseline uninfected children.

	Hookworm infection: baseline uninfected children						
	Baseline n/N (%)	3 months n/N (%)	7 monthsn/N (%)	crude % point risk difference with placebo (95% CI)	aOR^1^ (N = 982)	95% CI	P value
Placebo	0/280 (0.0)	26/214 (12.1)	23/194 (11.9)		1 (ref)		
Any fortified rice	0/854 (0.0)	97/576 (16.8)	125/591 (21.2)	+ 9.3% (2.9 to 14.7)	1.86	1.17 to 2.95	0.009
- UltraRice_ original	0/280 (0.0)	34/207 (16.4)	56/228 (24.6)	+ 12.7% (5.3 to 19.8)	1.89	1.06 to 3.37	0.032
- UltraRice_improved	0/297 (0.0)	39/208(18.8)	41/188 (21.8)	+ 10.0% (2.5 to 17.4)	1.91	1.12 to 3.26	0.018
- NutriRice	0/277 (0.0)	24/161 (14.9)	28/175 (16.0)	+ 4.1% (-2.9 to 11.4)	1.74	0.63 to 4.81	0.284

^1^ From mixed modelanalysis, adjusted for sex, age (in quartiles) and baseline prevalence of hookworm at the school.

**Table 4:** Effect of micronutrient fortified rice on hookworm infection risk of baseline uninfected children, stratified by baseline school hookworm prevalence.

	Hookworm infection risk by school prevalence category					
	Baseline prevalence < 15%					
	Baseline n/N (%)	3 months n/N (%)	7 months n/N (%)	aOR^1^ (N = 493)	95% CI	P value
Placebo	0/92(0.0)	15/82 (18.3)	4/74 (5.4)	1 (ref)		
Any fortified rice	0/477 (0.0)	35/312 (11.2)	44/311 (14.1)	0.96	0.62 to 1.47	0.840
- UltraRice_ original	0/87(0.0)	13/64 (20.3)	19/69 (27.5)	2.29	2.13 to 2.46	<0.001
- UltraRice_ improved	0/162(0.0)	11/116 (9.4)	10/91 (10.9)	0.82	0.49 to 1.37	0.454
- NutriRice	0/228(0.0)	11/132 (8.3)	15/151 (9.9)	0.73	0.53 to 1.03	0.070
	Baseline prevalence >15%					
	Baseline n/N (%)	3 months n/N (%)	7 months n/N (%)	aOR^1^ (N = 489)	95% CI	P value
Placebo	0/139(0.0)	11/132 (8.3)	19/120 (15.8)	1 (ref)		
Any fortified rice	0/377 (0.0)	62/264 (23.5)	81/280 (28.9)	2.94	1.82 to 4.77	<0.001
- UltraRice_ original	0/193(0.0)	21/143 (14.7)	37/159 (26.3)	1.82	1.33 to 2.49	<0.001
- UltraRice_ improved	0/135(0.0)	28/92 (30.4)	31/97 (32.0)	3.73	2.76 to 5.05	<0.001
- NutriRice	0/49(0.0)	13/29 (44.8)	13/24 (54.2)	8.65	6.59 to 11.34	<0.001

^1^From mixed model analysis, adjusted for sex and age (in quartiles).

Fecal calprotectin concentrations had increased after seven months in all groups (Table 5). Neither type of UltraRice had a significant effect on fecal calprotectin concentration compared to placebo. Calprotectin and hookworm infection were not associated at either baseline or at seven months.

**Table 5:** Effects of UltraRice_original and UltraRice_improved on prevalence of elevated fecal calprotectin (>50 mg/kg).

	Fecal calprotectin >50 mg/kg				
	Baseline n/N (%)	7 months n/N (%)	aOR^1^	95% CI	P value
Placebo	13/124 (10.5)	39/101 (38.6)	1 (ref)		
Any fortified rice	20/240 (8.3)	78/243 (32.1)	0.69	0.46 to 1.02	0.065
- UltraRice_original	7/117 (6.0)	39/121 (32.2)	0.74	0.32 to 1.72	0.481
- UltraRice_improved	13/123 (10.6)	39/122 (32.0)	0.66	0.29 to 1.51	0.324

^1^ From mixed model analyses, adjusted for sex, age (in quartiles), baseline calprotectin >50 mg/kg yes/no, N = 330

## Discussion

To our knowledge, this is the first study to show a significantly increased risk of hookworm infection in children receiving multi-micronutrient fortified rice. Negative effects of iron supplements on hookworm infection prevalence have been reported in adults, although this appeared to be a transient effect[20]. In our study, the effect of fortified rice on hookworm prevalence was modified by the baseline prevalence of hookworm at the schools, indicating that infection pressure is of importance.

The strong modifying effect that school hookworm prevalence had on the hookworm infection risk effects of fortified rice warrants caution when implementing micronutrient supplementation strategies in endemic areas. Even though all schools were in the same province, we found large differences in baseline hookworm prevalence across schools. Our results show that even within the same province, large regional differences can exist in the health effects of consumption of multi-micronutrient fortified rice by school children. Public health policies aimed at improving child micronutrient status should take hookworm infection risk into account, as hookworms are known to induce iron deficiency through blood loss[21]. Our results suggest that aside from anthelminthic treatment at schools, the abundance of hookworm eggs and larvae in the environment needs to be reduced to safeguard the children from (re)infection.

The increase in hookworm prevalence in all groups was surprising, given the anthelminthic treatment that was provided after baseline measurements. However, mebendazole has recently been shown to be significantly less effective than albendazole against hookworm in Cambodia[22]. The overall increase of infection might be a seasonal effect. It was also unexpected that 52 children who were infected at three months seemed uninfected at seven months, since no treatment was given at the schools between those time points. We suspect these to be false negatives; the Kato Katz diagnostic method is not very sensitive for low intensity hookworm infections [23]. Treatment received outside of school or study programs could also explain this observation. These 52 children were randomly distributed over all intervention groups, and definition of these 52 cases as ‘positive’ for hookworm at seven months did not change the findings.

The low number of schools per study group is an important limitation of this study, especially because the prevalence at school level was a large effect modifier. Moreover, the unexpected high variability in baseline hookworm infection prevalence creates imbalance between schools and study groups. The estimated odds ratios were exceptionally high for UltraRice_original in the stratum with low baseline prevalence and for NutriRice in the stratum with baseline prevalence above 15%, however for both these estimates only one school was included. We therefore emphasize the need for better-powered trials to confirm our findings. Also, the amounts of rice consumed by the children were not recorded in this study. Because the three types of fortified rice differed on content of several micronutrients, we cannot draw conclusions about causation of the increased hookworm risk by any one nutrient or amount thereof.

We did not find an effect of micronutrient-fortified rice on intestinal inflammation, measured as fecal calprotectin. Increases in fecal calprotectin after iron supplementation or fortification were found in studies in Ivorian children and Kenyan infants, but not in South African children [12, 24, 25]. Hookworm and calprotectin were not associated with each other at either time point. While calprotectin is considered to be a marker for intestinal inflammation in general, it is mainly derived from neutrophils[19]. Despite hookworms causing mucosal damage, a lack of association between hookworm and neutrophil abundance is to be expected as hookworms express a neutrophil inhibiting factor, thereby perhaps dampening intestinal inflammation[26].

A 2013 systematic review on effects of (multi-) micronutrient fortification studies showed positive effects on micronutrient status but acknowledged the paucity of studies with other health outcomes[27]. A recent study from Vietnam found a large reduction in hookworm prevalence after iron and folic acid supplementation[28]. However, this study did not include a control group. A meta-analysis of (multi-)micronutrient supplementation or fortification on helminth infection risk showed a non-significant increased risk after iron supplementation but a protective effect of multi-micronutrient supplementation[7]. However, when we repeated this meta-analysis including only hookworm as outcome, a non-significant increase in hookworm infection risk after multi-micronutrients was observed. In addition to strengthening the evidence for an increase in hookworm infection risk after multi-micronutrient fortification, we here show that local prevalence also plays an important role in this effect.

During systemic inflammatory (acute phase) responses, micronutrients such as iron and zinc are withheld from the human circulation[29]. This is considered a strategy against parasitic organisms who are also in need of these scarce micronutrients, a phenomenon described as ‘nutritional immunity’[30]. Within the context of withholding micronutrients from parasites, supplementation of these nutrients could override nutritional immunity and enhance the infection. This is especially the case for enteric parasites: with increasing micronutrient content of the gut, feeding the parasite instead of the host might become a serious risk. However, as hookworms are not known to feed on luminal contents, an increase in micronutrient concentration of mucosal tissue and blood would be needed to actually feed this parasite. Aside from risk of parasitic infection, the increased micronutrient availability in the gut lumen might influence intestinal microflora composition, which can in turn have a wide range of health consequences [12, 31].

Together, our results raise questions about possible negative health outcomes of micronutrient fortification of staple foods in hookworm endemic areas. Special attention might be warranted for so-called ‘home fortification’ of complementary foods with micronutrient powders. A literature search for the effect of home fortification with micronutrient powders on hookworm infection returned no published papers. We believe further research in this area is urgently needed.

The merits of micronutrient repletion should be weighed carefully against its possible risks. This might need to be considered for every region separately, taking into account local infection prevalence, severity of micronutrient deficiencies and other possible factors of influence. Pairing micronutrient supplementation with vigorous efforts to reduce hookworm infection risk, by frequent administration of anthelminthics and sanitation and hygiene interventions may circumvent the increased risk of hookworm infection, however this would need to be addressed by further studies.

## Supporting Information

S1 CONSORT Checklist CONSORT checklist for the reporting of cluster trials. (DOCX)

S1 Fig Hookworm infection prevalence at each school, by study group. N is the number of children at the school for which a baseline hookworm diagnosis was available.
(TIF)

S1 Protocol The original protocol of the FORISCA study. (PDF)
