# Supplementary material for: Micronutrient-Fortified Rice Can Increase Hookworm Infection Risk: A Cluster Randomized Trial
Source: PLoS One. 2016 Jan 6;11(1):e0145351. doi: 10.1371/journal.pone.0145351 (PMC4703301; doi:10.1371/journal.pone.0145351)
Supplement: S1 CONSORT Checklist — (DOCX) [file pone.0145351.s001.docx]

**Table 1: CONSORT 2010 checklist of information to include when reporting a cluster randomised trial**

| Section/Topic | Item No | Standard Checklist item | Extension for cluster designs | Page No * |
| --- | --- | --- | --- | --- |
| Title and abstract | | | |  |
|  | 1a | Identification as a randomised trial in the title | Identification as a cluster randomised trial in the title | 1 |
|  | 1b | Structured summary of trial design, methods, results, and conclusions (for specific guidance see CONSORT for abstracts)^[[1]](#endnote-1),^^[[2]](#endnote-2)^ | See table 2 | 2 |
| Introduction | | | |  |
| Background and objectives | 2a | Scientific background and explanation of rationale | Rationale for using a cluster design | 4  *A cluster-randomization was chosen because the schools had one kitchen each, and separate preparations of school meals were not feasible.* |
|  | 2b | Specific objectives or hypotheses | Whether objectives pertain to the the cluster level, the individual participant level or both | 3  *To examine the effect of the introduction of fortified rice within the World Food Program (WFP) school meal program on nutritional status and health, a large cluster-randomized, placebo controlled trial with three types of micronutrient-fortified rice was conducted. Here we report on effects of the introduction of fortified rice on hookworm infection and local intestinal inflammation.* |
| Methods | | | |  |
| Trial design | 3a | Description of trial design (such as parallel, factorial) including allocation ratio | Definition of cluster and description of how the design features apply to the clusters | 3-4  *The clusters were 16 primary schools in rural Kampong Speu province, of which four were randomly selected for each study group.* *The clusters were 16 primary schools in rural Kampong Speu province, of which four were randomly selected for each study group.* |
|  | 3b | Important changes to methods after trial commencement (such as eligibility criteria), with reasons |  | n.a. |
| Participants | 4a | Eligibility criteria for participants | Eligibility criteria for clusters | 3-4  *Schools were eligible if they participated in the WFP school meal program and all children were served breakfast daily. In total 18 schools were eligible, two were excluded because of the number of school children (one school had double the number of school children (N=1200) as the other schools, and one school had <100 school children, whereas for biochemical determination of micronutrient status a minimum of 125 school children was required per school).* |
|  | 4b | Settings and locations where the data were collected |  | 3  *16 primary schools in rural Kampong Speu province* |
| Interventions | 5 | The interventions for each group with sufficient details to allow replication, including how and when they were actually administered | Whether interventions pertain to the cluster level, the individual participant level or both | 3-4  *A cluster-randomization was chosen because the schools had one kitchen each, and separate preparations of school meals were not feasible. The trial took place from November 2012 to June 2013. The clusters were 16 primary schools in rural Kampong Speu province, of which four were randomly selected for each study group.*  *Children received one type of fortified rice or placebo (unfortified white rice) six days per week for six months.* |
| Outcomes | 6a | Completely defined pre-specified primary and secondary outcome measures, including how and when they were assessed | Whether outcome measures pertain to the cluster level, the individual participant level or both | 4-5  *The primary outcome for this report is hookworm infection, which was the main intestinal parasite found in this population. Fresh stool samples were collected and analyzed by Kato-Katz technique at baseline (before treatment), three months and seven months (one month after the intervention ended) to determine hookworm infection. Parasite diagnosis was performed by the National Center for Parasitology, Entomology and Malaria control (CNM), Phnom Penh, Cambodia, and recorded as eggs per gram of feces. For a subgroup of 330 children, at baseline and after seven months stool samples were frozen (-20° C) and sent to the Institute for Tropical Medicine in Antwerp, Belgium where our secondary outcome calprotectin was measured by ELISA (Calpro AS, Norway)* |
|  | 6b | Any changes to trial outcomes after the trial commenced, with reasons |  | n.a. |
| Sample size | 7a | How sample size was determined | Method of calculation, number of clusters(s) (and whether equal or unequal cluster sizes are assumed), cluster size, a coefficient of intracluster correlation (ICC or *k*), and an indication of its uncertainty | 4  *The clusters were 16 primary schools in rural Kampong Speu province, of which four were randomly selected for each study group In total 18 schools were eligible, two were excluded because of the number of school children (one school had double the number of school children (N=1200) as the other schools, and one school had <100 school children, whereas for biochemical determination of micronutrient status a minimum of 125 school children was required per school).* |
|  | 7b | When applicable, explanation of any interim analyses and stopping guidelines |  | n.a. |
| Randomisation: | | | |  |
| Sequence generation | 8a | Method used to generate the random allocation sequence |  | *5*  *Three different randomizations, combining different schools to one intervention, were separately generated based on a list number of children per school by iteration to fit the predefined criteria of group size (within 10% of the mean).* |
|  | 8b | Type of randomisation; details of any restriction (such as blocking and block size) | Details of stratification or matching if used | n.a. |
| Allocation concealment mechanism | 9 | Mechanism used to implement the random allocation sequence (such as sequentially numbered containers), describing any steps taken to conceal the sequence until interventions were assigned | Specification that allocation was based on clusters rather than individuals and whether allocation concealment (if any) was at the cluster level, the individual participant level or both | 5  *…allocated each group of schools to an intervention arm. To further assure blinding, each intervention arm of 4 schools was split into two groups of two schools, each given a letter code (A – H). The entire research team and all participants and caregivers were blinded to the allocation.* |
| Implementation | 10 | Who generated the random allocation sequence, who enrolled participants, and who assigned participants to interventions | Replace by 10a, 10b and 10c |  |
|  | 10a |  | Who generated the random allocation sequence, who enrolled clusters, and who assigned clusters to interventions | 5  *A researcher not involved in the field work (MAD) blindly picked one of the three randomizations, and allocated each group of schools to an intervention arm.* |
|  | 10b |  | Mechanism by which individual participants were included in clusters for the purposes of the trial (such as complete enumeration, random sampling) | 4  *The clusters were 16 primary schools*  *Children received one type of fortified rice or placebo (unfortified white rice) six days per week for six months.*  *+ figure 1* |
|  | 10c |  | From whom consent was sought (representatives of the cluster, or individual cluster members, or both), and whether consent was sought before or after randomisation | 4  *Written informed consent of at least one parent was obtained prior to the study.* |
|  |  |  |  |  |
| Blinding | 11a | If done, who was blinded after assignment to interventions (for example, participants, care providers, those assessing outcomes) and how |  | 5  *The entire research team and all participants and caregivers were blinded to the allocation. The code was only known to one person with WFP, responsible to allocate the correct type of rice to the right school. The rice packaging was coded with the letter allocated during the randomization (A – H) and did not contain the name of the rice type.* |
|  | 11b | If relevant, description of the similarity of interventions |  | Table 1 |
| Statistical methods | 12a | Statistical methods used to compare groups for primary and secondary outcomes | How clustering was taken into account | 5  *We accounted for the school clusters by including school baseline prevalence of hookworm, which differed per school, as a continuous covariate.* |
|  | 12b | Methods for additional analyses, such as subgroup analyses and adjusted analyses |  | 5  *We focused the analysis on the effect of the intervention on hookworm infection in children who were uninfected at baseline, in order to estimate new infection rate.*  *Covariates in all models were sex and age in quartiles. Effect modification was examined by introducing interaction terms into the model, when these showed significant effects (p <0.05), we stratified the analysis.* |
| Results | | | |  |
| Participant flow (a diagram is strongly recommended) | 13a | For each group, the numbers of participants who were randomly assigned, received intended treatment, and were analysed for the primary outcome | For each group, the numbers of clusters that were randomly assigned, received intended treatment, and were analysed for the primary outcome | Figure 1 |
|  | 13b | For each group, losses and exclusions after randomisation, together with reasons | For each group, losses and exclusions for both clusters and individual cluster members | Figure 1 |
| Recruitment | 14a | Dates defining the periods of recruitment and follow-up |  | 4  *The trial took place from November 2012 to June 2013.* |
|  | 14b | Why the trial ended or was stopped |  | n.a. |
| Baseline data | 15 | A table showing baseline demographic and clinical characteristics for each group | Baseline characteristics for the individual and cluster levels as applicable for each group | Table 2 |
| Numbers analysed | 16 | For each group, number of participants (denominator) included in each analysis and whether the analysis was by original assigned groups | For each group, number of clusters included in each analysis | Figure 1 |
| Outcomes and estimation | 17a | For each primary and secondary outcome, results for each group, and the estimated effect size and its precision (such as 95% confidence interval) | Results at the individual or cluster level as applicable and a coefficient of intracluster correlation (ICC or k) for each primary outcome | Table 3-5 |
|  | 17b | For binary outcomes, presentation of both absolute and relative effect sizes is recommended |  | Table 3 |
| Ancillary analyses | 18 | Results of any other analyses performed, including subgroup analyses and adjusted analyses, distinguishing pre-specified from exploratory |  | Table 4-5 |
| Harms | 19 | All important harms or unintended effects in each group (for specific guidance see CONSORT for harms^[[3]](#endnote-3)^) |  | Table 3-4 (primary outcome is a harm), further harms n.a. |
| Discussion | | | |  |
| Limitations | 20 | Trial limitations, addressing sources of potential bias, imprecision, and, if relevant, multiplicity of analyses |  | 8  *The low number of schools per study group is a limitation of this study, because the prevalence at school level was a large effect modifier. Because the three types of fortified rice differed on content of several micronutrients, we cannot draw conclusions about causation of the increased hookworm risk by any one nutrient or amount thereof.*  *The increase in hookworm prevalence in all groups was surprising, given the anthelminthic treatment that was provided after baseline measurements. However, albendazole given as a single dose was shown to have a low cure rate in a recent study in Lao PDR (21). The overall increase of infection might be a seasonal effect. It was also unexpected that 52 children who were infected at three months seemed uninfected at seven months, since no treatment was given at the schools between those time points. We suspect these to be false negatives; low intensity infections can be difficult to diagnose microscopically.* |
| Generalisability | 21 | Generalisability (external validity, applicability) of the trial findings | Generalisability to clusters and/or individual participants (as relevant) | 8  *The strong modifying effect that school hookworm prevalence had on the hookworm infection risk effects of fortified rice warrants caution when implementing micronutrient supplementation strategies in endemic areas. Even though all schools were in the same province, we found large differences in baseline hookworm prevalence across schools. Our results show that even within the same province, large regional differences can exist in the health effects of consumption of multi-micronutrient fortified rice by school children.* |
| Interpretation | 22 | Interpretation consistent with results, balancing benefits and harms, and considering other relevant evidence |  | 9  *Together, our results raise questions about possible negative health outcomes of micronutrient fortification of staple foods in hookworm endemic areas. Special attention might be warranted for so-called ‘home fortification’ of complementary foods with micronutrient powders. A literature search for the effect of home fortification with micronutrient powders on hookworm infection returned no published papers. We believe further research in this area is urgently needed.*  *The merits of micronutrient repletion should be weighed carefully against its possible risks. This might need to be considered for every region separately, taking into account local infection prevalence, severity of micronutrient deficiencies and other possible factors of influence. Pairing micronutrient supplementation with vigorous efforts to reduce hookworm infection risk, by frequent administration of albenzadole and sanitation and hygiene interventions may circumvent the increased risk of hookworm infection, however this would need to be addressed by further studies.* |
| Other information | | |  |  |
| Registration | 23 | Registration number and name of trial registry |  | *This trial is registered with ClinicalTrials.gov under number NCT01706419.* |
| Protocol | 24 | Where the full trial protocol can be accessed, if available |  | *ClinicalTrials.gov* |
| Funding | 25 | Sources of funding and other support (such as supply of drugs), role of funders |  | *This study was funded by* ***USDA/FAS, WFP-DSM consortium and IRD.*** |

** Note: page numbers optional depending on journal requirements*

**Table 2: Extension of CONSORT for abstracts**1**^,^**2 **to reports of cluster randomised trials**

| Item | Standard Checklist item | Extension for cluster trials |
| --- | --- | --- |
| Title | Identification of study as randomised | Identification of study as cluster randomised |
| Trial design | Description of the trial design (e.g. parallel, cluster, non-inferiority) |  |
| Methods |  |  |
| Participants | Eligibility criteria for participants and the settings where the data were collected | Eligibility criteria for clusters |
| Interventions | Interventions intended for each group |  |
| Objective | Specific objective or hypothesis | Whether objective or hypothesis pertains to the cluster level, the individual participant level or both |
| Outcome | Clearly defined primary outcome for this report | Whether the primary outcome pertains to the cluster level, the individual participant level or both |
| Randomization | How participants were allocated to interventions | How clusters were allocated to interventions |
| Blinding (masking) | Whether or not participants, care givers, and those assessing the outcomes were blinded to group assignment |  |
| Results |  |  |
| Numbers randomized | Number of participants randomized to each group | Number of clusters randomized to each group |
| Recruitment | Trial status^^[[4]](#footnote-1)^^ |  |
| Numbers analysed | Number of participants analysed in each group | Number of clusters analysed in each group |
| Outcome | For the primary outcome, a result for each group and the estimated effect size and its precision | Results at the cluster or individual participant level as applicable for each primary outcome |
| Harms | Important adverse events or side effects |  |
| Conclusions | General interpretation of the results |  |
| Trial registration | Registration number and name of trial register |  |
| Funding | Source of funding |  |
|  |  |  |

**REFERENCES**

1. Hopewell S, Clarke M, Moher D, Wager E, Middleton P, Altman DG, et al. CONSORT for reporting randomised trials in journal and conference abstracts. *Lancet* 2008, 371:281-283 [↑](#endnote-ref-1)
2. Hopewell S, Clarke M, Moher D, Wager E, Middleton P, Altman DG at al (2008) CONSORT for reporting randomized controlled trials in journal and conference abstracts: explanation and elaboration. *PLoS Med* 5(1): e20 [↑](#endnote-ref-2)
3. Ioannidis JP, Evans SJ, Gotzsche PC, O'Neill RT, Altman DG, Schulz K, Moher D. Better reporting of harms in randomized trials: an extension of the CONSORT statement. *Ann Intern Med* 2004; 141(10):781-788. [↑](#endnote-ref-3)
4. Relevant to Conference Abstracts [↑](#footnote-ref-1)
