# Supplementary figures and images for: Micronutrient-Fortified Rice Can Increase Hookworm Infection Risk: A Cluster Randomized Trial
Source: PLoS One. 2016 Jan 6;11(1):e0145351. doi: 10.1371/journal.pone.0145351 (PMC4703301; doi:10.1371/journal.pone.0145351)

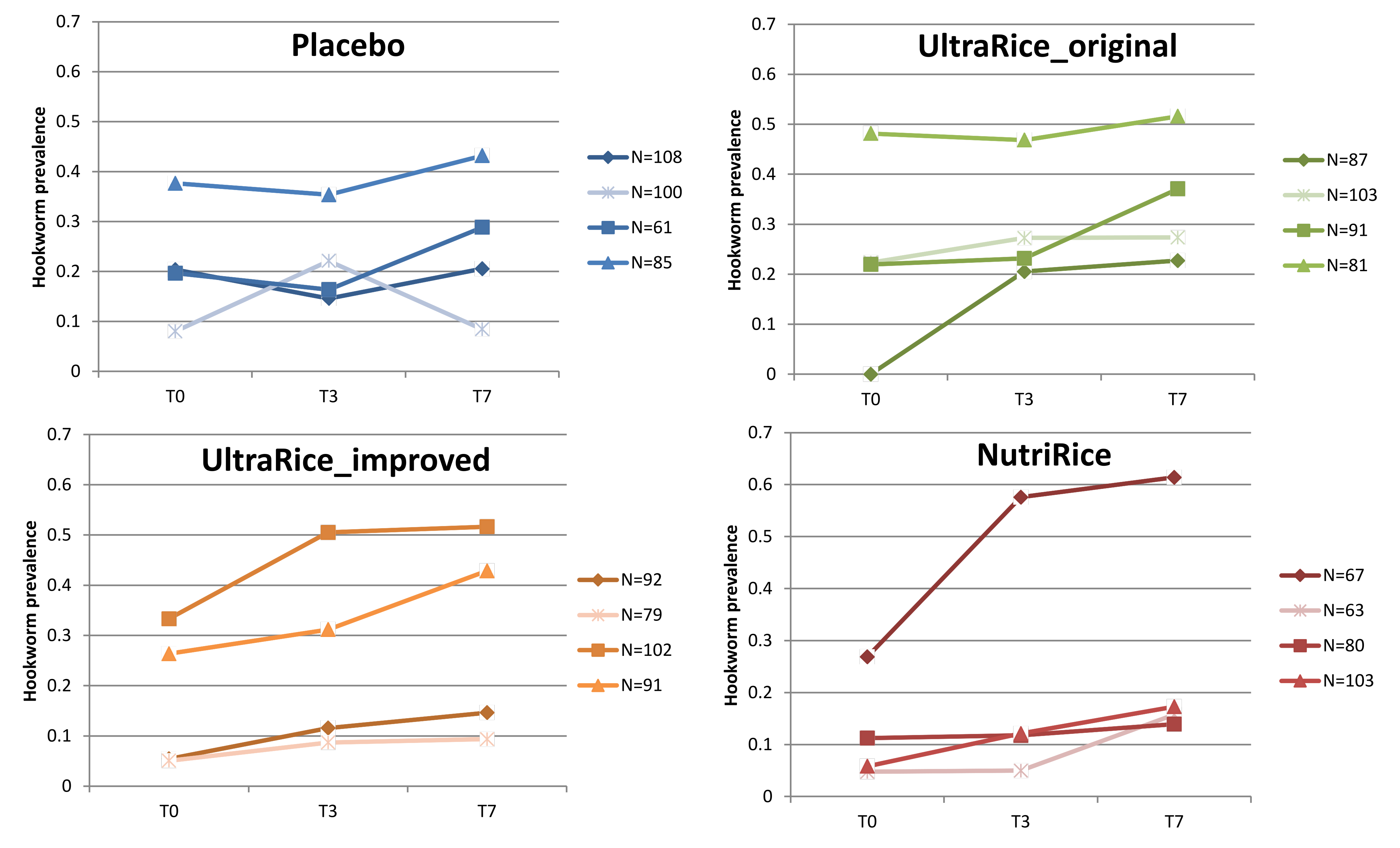

Supplement: S1 Fig — N is the number of children at the school for which a baseline hookworm diagnosis was available. (TIF) [file pone.0145351.s002.tif]
