# Supplementary material for: Micronutrient-Fortified Rice Can Increase Hookworm Infection Risk: A Cluster Randomized Trial
Source: PLoS One. 2016 Jan 6;11(1):e0145351. doi: 10.1371/journal.pone.0145351 (PMC4703301; doi:10.1371/journal.pone.0145351)
Supplement: S1 Protocol — (PDF) [file pone.0145351.s003.pdf]

FORISCA PROJECT

Study protocol
April 2012

CONFIDENTIAL

Institute for Research for Development (IRD)
PATH
World Food Program Cambodia (WFP)

Introduction of Fortified Rice for School Meals in Cambodia (FORISCA) to Improve Health and Development of Cambodian School Children

April 2012

Submitted to the Ethical Committee of the Ministry of Health, Kingdom of Cambodia

Executive summary

Micronutrient deficiencies are a major health problem in many developing countries, including Cambodia. Micronutrient deficiency results in poor health, with a much higher risk for mortality in vulnerable groups such as pregnant women and young infants, and delayed physical and cognitive development, resulting in retarded growth and stunting. It thereby prevents children from reaching their full potential as micronutrients play an important role in children's cognitive and motor development.

The UN World Food Program (WFP) provides a daily school meal to ~500,000 school children in Cambodia. The main objective of the school meal program is to increase school attendance. Although the school meal provides salt fortified with iodine, and vegetable oil fortified with vitamin A, the school meal provides an excellent opportunity to improve micronutrient status of school children by providing them with rice fortified with vitamins and minerals such as zinc, vitamin A, iron, folic acid and vitamin B_{12}. This is likely to reduce the prevalence of anemia in school children and improve health and school performance. Anemia is a major problem in school children in Cambodia. A recent study by WFP showed that more than half of the school children were anemic.

Rice fortification has been shown to be safe and effective in improving micronutrient status in many studies. However, most studies have been done in Latin America, Africa and India. There are no studies available from Cambodia, except for an acceptability study of fortified rice conducted by WFP and the Institute of Research for Development (IRD) in 2010 in Kampung Speu. This acceptability study showed that fortified rice has an excellent acceptability among school children, parents and teachers. However, there is no data available on whether fortified rice can reduce the prevalence of anemia in school children in Cambodia, nor whether there will be additional benefits, such as better learning capability of school children or fewer days missed due to better health.

To inform WFP, Cambodian policy and the public on the potential benefits of fortified rice on health and development for Cambodian school children, an intervention study will be conducted in Kampung Speu province during the 2012 – 2013 school year. The study will be conducted by IRD, PATH and WFP. Sixteen (16) schools will be selected to receive either the normal rice provided by the WFP school meal program, or fortified rice instead of normal rice. Three (3) different types of fortified rice will be tested, to identify the best type of fortified rice. In addition, 4 schools with no school meal program will be selected to serve as control. School attendance and morbidity will be followed in all children in the participating schools over the whole school year. Biochemical indicators of micronutrient status will be determined in a subgroup of children (25% of the children), which requires collection of blood, urine and stool samples.

The study will be submitted to the Ethical Committee of the Ministry of Health for approval, and the Ethical Review Board of PATH, USA. Furthermore, approval will be obtained from the Ministries of Health, Education and Planning prior to the commencement of the study.

Potential benefits of the study includes the immediate treatment of school children found to have severe anemia (hemoglobin < 70 g/L) and improved health and cognitive development for all children receiving fortified rice. Potential disadvantages and risks of the study include side effects of blood taking in the subgroup of children and the disruption of classes for a few days during the data collection.

If the results of the study show improved health and cognition for school children receiving fortified rice, provision of fortified rice to all 500,000 Cambodian school children taking part in the WFP school meal program in Cambodia could become a priority.

FORISCA PROJECT DESCRIPTION

1.1 Introduction

More people are affected by anemia and iron deficiency (ID) than by any other micronutrient deficiency, with an estimated 1.6 billion people being anemic and even more people currently having insufficient iron stores[1]. Iron deficiency will eventually lead to iron-deficiency anemia (IDA). Indeed, the World Health Organization (WHO) estimates that roughly twice as many people are affected by iron deficiency than by IDA[2]. However, before anemia occurs, iron deficiency is already affecting other functions, such as the immune system and the nervous system, leading to reduced immunocompetence, decreased physical activity and cognitive impairment[3]. On the other hand, many cases of anemia are not due to ID but to other causes such as nutritional deficiencies other than iron (e.g. deficiency of vitamin B12, folic acid or vitamin A), genetic traits (hemoglobinopathies such as sickle-cell or thalassaemia) or chronic inflammation. Besides iron, deficiencies of other micronutrients such as vitamin A, zinc, folic acid and iodine are often highly prevalent in developing countries, including Cambodia[4]. Indeed, micronutrient deficiencies often coexist, and finding only a single micronutrient deficiency in one subject is rather the exception than a rule[5]. Many of these micronutrients function in the same biochemical pathway. Therefore, providing multiple micronutrients instead of only one works synergistically[6], as for example vitamin A stimulates the production of new red blood cells, improving the utilization of the provided iron[7, 8], and both iron and vitamin A deficiency might aggravate iodine deficiency[9].

Deficiency of many of these micronutrient deficiencies can also result in delayed physical and cognitive development, resulting in retarded growth and stunting[10], and in preventing children from reaching their full potential as micronutrients play an important role in children's cognitive and motor development[11]. Indeed, children living in areas affected by severe iodine deficiency disorder (IDD) may have an intelligence quotient (IQ) of more than 12 points below that of non-iodine-deficient areas[12]. Besides iodine, deficiencies of iron and zinc have been associated with impaired psycho-motor development and cognitive function[13, 14]. Iron deficiency in school-aged children, even before the onset of anemia, may affect learning abilities[15] and zinc deficiency has been linked with low activity and depressed motor development among the most vulnerable children[16]. As deficiency of several micronutrients has been implicated in impaired cognitive and motor performance and development, correction of a single deficiency may not be enough to substantially improve cognitive performance.

Another factor affecting nutritional status, health and cognitive function is parasite infestation. The exact role of intestinal parasite infection in cognitive development is unclear, as most data is based on cross-sectional surveys which might be biased by factors such as poverty and poor nutritional status[17]. But intestinal parasites reduce the available iron in the gut, and cause local inflammation, thereby causing a loss of nutrients. And frequent absenteeism from school because of illness due to intestinal parasites will affect

educational achievements for sure[18]. For Cambodia, there is no national data on the prevalence of intestinal parasite infection, but small studies show an infestation rate of >50%[19]. Currently, >75% of the Cambodian school children receive deworming treatment every 6 months, but this might not be often enough to make a lasting impact. Recently, we showed in Vietnam that improving micronutrient status of school children through fortified biscuits markedly reduced the re-infection rate with parasites. Parasite infection rates in children receiving deworming, but no micronutrients were back to baseline prevalence after 6 months, whereas in children receiving both, re-infections rates were lower[20].

1.2 **Situation in Cambodia**

While Cambodia has seen considerable improvement in access to food and nutritional status since it emerged from civil war in the 1990s, many people continue to suffer from malnutrition and food insecurity and related indicators remain among the lowest in the region. Cambodia ranks 124th of the 169 countries in the 2010 Human Development Index rankings, with 26 percent of its 14.8 million people living on less than $1.25 per day. Over 2.5 million people live in extreme poverty and face serious food deprivation, and the poverty rate for children under five years of age is 38%. The worldwide increase in food prices has also affected Cambodia, accentuating the vulnerability of low-income households. Indeed, child undernutrition in Cambodia is high, with Cambodia being ranked among the 36 countries with the highest burden of child undernutrition[21].

A recent survey highlighted the need for urgent action, with 11 percent of children under the age of five having severe acute malnutrition, 29 percent being classified as underweight, and 40 percent of the children being stunted. Worryingly, little improvement has been seen in the rates of underweight , stunted, and wasted children since 2005. Therefore, new strategies to combat micronutrient deficiencies in Cambodia are therefore urgently needed.

Proportion of undernourished children in Cambodia in the National Demographic and Health Surveys of 2005 and 2010.

**Year**	**Underweight (%)** Weight for age	**Stunted (%)** Height for age	**Wasted (%)** Weight for height
**2005**	28	43	8
**2010**	28	40	11

The most commonly consumed foods among children in Cambodia are rice and fish, followed by sweets. Few children are eating important sources of protein, energy and vitamins and minerals such as legumes and nuts (10%), oils and fats (23.1%), and animal products (13.9%). Therefore, micronutrient deficiencies are likely to be very prevalent in Cambodia. This is supported by the limited data available on micronutrient deficiencies in Cambodian children. The most recent data available on vitamin A deficiency, from 2000, found that 22 percent of rural children were deficient, while the recent Cambodia Demographic and Health Survey 2010 showed that anemia rates among children under age five have only shown slight improvement (from 62 percent in 2000 to 55 percent in 2010).

Despite the dearth of data, given the high rates of undernutrition and stunting, micronutrient deficiencies are highly prevalent in Cambodia.

The World Food Program (WFP) school feeding program, supported in part through the McGovern Dole program, provides a daily breakfast using a standard nutritionally optimized WFP food ration to almost 500,000 school children in Cambodia. The program is an existing large-scale McGovern-Dole supported intervention with well-established relationships with the government and delivery infrastructure. The program encourages poor and hungry children to come to school and affords them an opportunity to concentrate on their study. In addition, poorer students, particularly girls in the upper primary grades, are provided with a family take home ration.

An assessment of the program found that school feeding was a strong incentive for parents to send their children to school, but only while schools benefited from the program. The evaluation also noted that inclusion of fortified rice could benefit the program if it could reduce the prevalence of iron-deficiency anemia in both sexes. One of the evaluation's final recommendations was that the country office should design and implement modalities for including fortified food in its school feeding program[22].

Fortification of staple foods is a cost-effective tool to improve micronutrient status of populations at risk for micronutrient deficiencies. Of the tools currently available to alleviate nutritional deficiencies among vulnerable groups, food fortification is the most promising and cheapest[23]. A number of fortified food products are available in Cambodia. However, other than iodized salt, none have reached any significant scale within the country. Rice is the main staple for Cambodians, accounting for 70 percent of daily calorie intake[24]. Per capita rice consumption was 189 kilograms in 2005, which is among the highest intakes of rice in the world. Given this very high consumption rate, the potential impact of fortifying rice in the country could be huge. The current intervention study aims to show the benefits of introducing fortified rice in the regular WFP school meal program on health and development of Cambodian school children.

2.1 Objectives and hypotheses

Objectives

The main objective of the intervention is to measure the impact of introducing fortified rice in the regular WFP school feeding program on several outcomes, namely:

1. Prevalence of anemia
2. Anthropometry (weight, height, Body Mass Index)
3. Prevalence of micronutrient deficiency (vitamin A, iron, zinc, vitamin B12 and iodine)
4. Cognitive function (as measured with Raven's Colored Matrices and other tests)
5. School attendance (as measured by days of absence due to illness)
6. Parasite infestation and gut flora
7. Immune function (as measured by morbidity recall and *ex vivo* cytokine responses)

Main objectives are objective 1 – 5, whereas objective 6 and 7 are secondary objectives, meaning that these last objectives will be included when budget and logistics allow.

Hypothesis
The main hypothesis is that the introduction of fortified rice will decrease:

a. the prevalence of anemia (obj. 1)
b. the prevalence of micronutrient deficiencies (obj. 3; vitamin A, iron, zinc, vitamin B12 and iodine).

These are direct measurements of micronutrient status, and are sensitive to an intervention. Therefore, a smaller sample size is needed (see below). However, as micronutrient status affects several functional outcomes, the secondary hypothesis is that the improved micronutrient status will also result in better functional outcomes. Specifically, that introduction of fortified rice will result in

c. higher anthropometrical indices (obj. 2)
d. better cognitive function (obj. 4)
e. higher school attendance (obj.5).

These functional outcomes are affected by other factors as well, and therefore less sensitive to an intervention. For these outcomes, a bigger sample size is needed (see below).

In addition, it is hypothezed that consumption of rice fortified with multiple micronutrients will affect immune function, resulting

f. increased benefit of half-yearly deworming through lower parasite re-infestation rates (obj. 6)
g. Changes in gut flora, harvesting less pathogenic bacteria (obj. 6)
h. Increased *ex vivo* cytokine responses after stimulation (obj. 7)

As these outcomes require collection of biochemical data, the sample size for the primary hypothesis will be followed.

2.2 Participants, Design and Methods

The study will be a cluster randomized, double blinded, placebo-controlled large scale effectiveness study. Cluster units will be schools in Kampung Speu province, Cambodia, which will be randomized to different treatment (see below). The study sample will consist of school children aged 7-14 years enrolled in participating schools. KampUng Speu province has been selected because it includes schools that both do and do not received support from WFP for the school meals program, giving easy access to a control group. It is also the site of the recently conducted acceptability study which showed excellent acceptability of fortified rice. Moreover, it is not a risk area for flooding, minimizing the risk for logistical problems for the study. During the heavy floods of 2011, school meal programs in some schools in other provinces such as Siem Reap could only be started several months after the school year had started. Finally, it is located within easy access of Phnom Penh

which will facilitate monitoring and supervision of intervention activities and transport of biological samples.

<u>Site description</u>.

Kampung Speu is one of the 23 provinces of Cambodia, situated 60 km west of the capital Phnom Penh. Kampung Speu province is subdivided into 8 districts (Baset, Chbar Mon, Kong Pisei, Aural, Udong, Phnom Sruoch Samraong Tong, Thpong), with Kampung Speu town as provincial capital. The majority of the inhabitants are dependent on agriculture for their living, with rice being the main crop. The 2008 population census showed that 716,000 people lived in Kampung Speu, with an annual growth rate of 1.79 and a population density of just over 100 persons/km^2. Infant mortality in Kampung Speu is still high at 65 / 1000 live births, compared to 13 / 1000 live births in the capital Phnom Penh (2010 CDHS), although primary health care facilities are good, and 89% of children receive full vaccination coverage during their first year of life. Twenty five percent of the population of Kampung Speu is between 5 and 14 years of age, comprising 180,000 children.

<u>WFP SCHOOL MEAL PROGRAM 2008 – 2010 IN CAMBODIA</u>

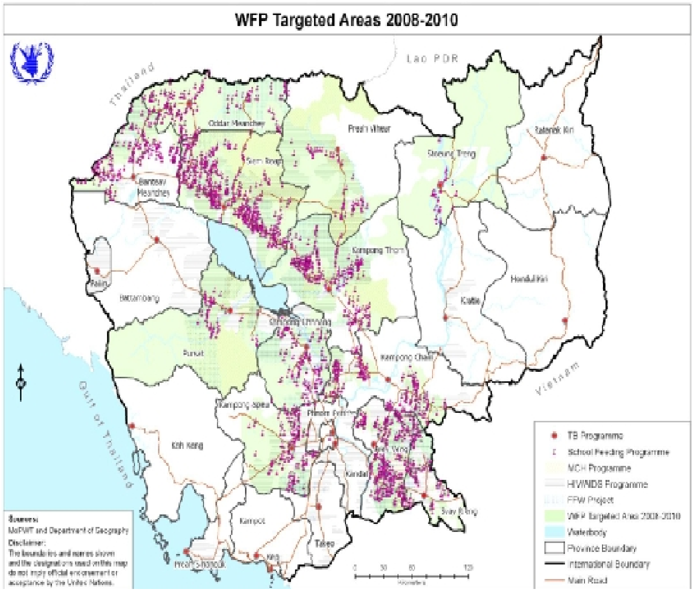

Study design

The effectiveness study will be conducted in <u>20</u> schools in Kampong Speu province (covering approximately 10,000 children), 16 of which participate in the WFP school meals program, and 4 which are not part of the school meal program. From the 309 schools in Kampung Speu, 90 schools receive school meals, of which 43 receive a school meal <u>in the morning only</u>, that is, there is no shift between morning and afternoon classes. From these 43 schools, <u>16</u> will be randomly selected for the study. In addition, from the 34 school not receiving school meals or a take-home ration, <u>4 schools</u> will be randomly selected. Selection

will be done by numbering each school and generation of 2 random lists of 16 schools and 4 schools respectively. The 16 schools receiving school meals will be randomized to receive either the normal school meal food basket (Control group), the original fortified rice (UR_original, the improved fortified rice (UR_improved), or the hot-extruded fortified rice (DSM). The 4 schools not receiving a school meal will serve as double control, to assess the impact of the normal school meal program, and the additional benefits of including fortified rice. The standard WFP school meal consists of rice, canned fish, vitamin-A fortified vegetable oil, yellow split peas, and iodized salt. In the 3 intervention groups with fortified rice, the normal rice shall be replaced with either original Ultra Rice or the improved Ultra Rice, or the fortified rice provided by DSM (NutriRice).

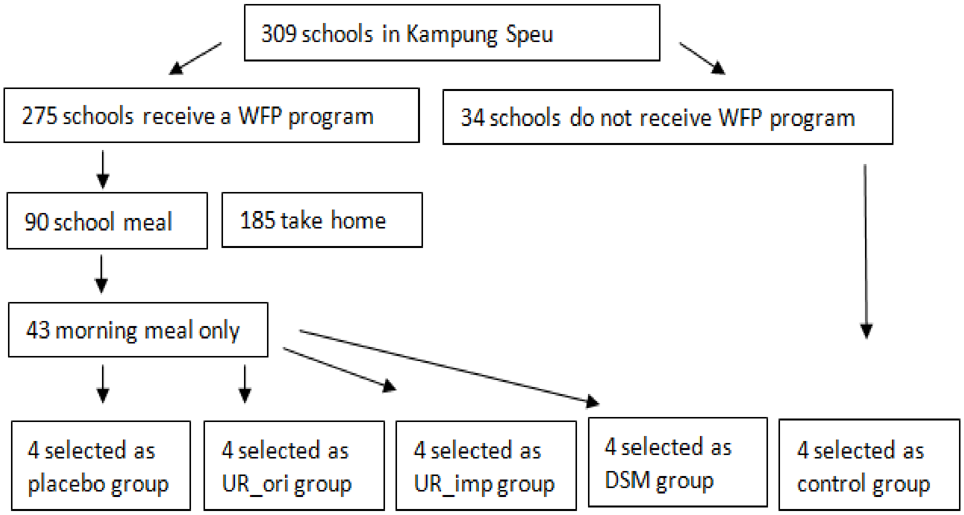

The intervention will run throughout the whole school year, from October 2012 until June 2013. The start of the intervention will coincide with the start of the school year, in October 2012, and run until the end of the school year, 10 months later. The study will involve four groups, each comprising four schools. WFP participating schools will be randomly selected to receive either A control group will be formed from an additional four schools not taking part in the WFP school meal program. During the whole intervention, breakfast will be served to school children each school day (Monday through Saturday).

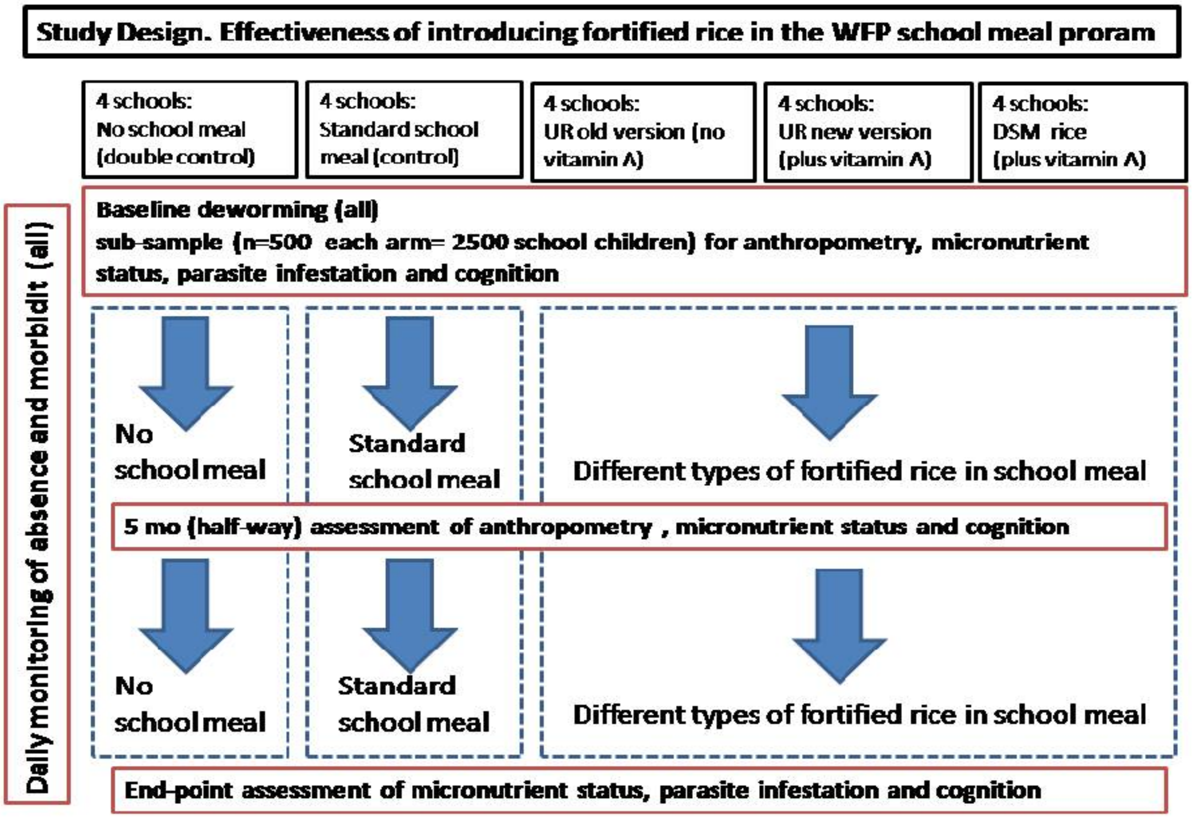

Composition of the fortified rice

The fortified rice will provide between 30% and 50% of the daily recommended allowance (RDA) of the included vitamins and minerals. In contrast to for example 200,000 IU high dose vitamin A supplements, which are being given to children between 1 and 5 years of age only every 6 months, the aim of fortified rice is to provide a low dose of vitamins and minerals every day. The advantage of this approach is that it is a more physiological approach to improve nutritional status. Moreover, there is no risk of toxicity by providing too much of a vitamin or mineral.

There are several methods for producing fortified rice. Two widely used methods are extrusion and waxing.

With the extrusion method, artificial rice kernels are made from a mixture of rice flour, a premix of vitamins and minerals and a binding agent. Extrusion can be cold (temperatures <70C) or hot (temperatures between 70C and 110C). The artificial rice kernels are mixed in a ratio of 1:100 with normal rice, making fortified rice suitable for consumption.
Waxing uses a coat with a premix of vitamins and minerals which are spread over the rice kernels. As washing of the rice before cooking might remove the wax coat, and with this, the vitamins and minerals, waxing is considered not suitable for SE Asia.

The FORISCA study will compare 3 different types of fortified rice, made with the extrusion method. The first type of rice is the original fortified rice from PATH. This type of rice does not contain vitamin A, and the fortified rice kernels are produced with cold extrusion. The second type of rice is the improved fortified rice kernel from PATH, which does contain vitamin A. As vitamin A and iron can react together, the vitamin A and iron have been coated before mixing with the rice flour. The temperature of extrusion is just above 70C, so the method of extrusion is somewhere between cold and hot. The final type of fortified rice kernel will be from DSM (NutriRice), which is a hot-extruded fortified rice kernel containing vitamin A and iron. For complete composition of the rice kernels, please see below.
The table below summarizes the aimed fortification levels for the different vitamins and minerals of the 3 types of fortified rice used in the FORISCA project, as well as the toxicity levels.

Vitamin / Mineral	UltraRice_orginal (cold extrusion)	UltraRice_ improved (in between cold and hot extrusion)	NutriRice (DSM) (hot extrusion)	Recommended Daily allowance for children 6 - 12 yrs	Toxicity levels	Factor toxicity / intake from fortified rice
Iron	4.0 mg	4.0 mg	4.0 mg	10 mg	20 mg/kg body weight	>50
Zinc	3.0 mg	3.0 mg	3.0 mg	6 mg	>50 mg/d	>15
Vitamin A	-	1000 IU	1000 IU	3300 IU	>200,000 IU*	>60
Thiamin (Vitamin B1)	300 µg	300 µg	300 µg	600 µg	Unknown, but >100 mg/d	>250
Folic Acid (Vitamin B9)	190 µg	190 µg	190 µg	400 µg	Unknown, but >10,000 ug/day	>50
Niacinamide (Vitamin B2)	4 mg	4 mg	4 mg	8 mg	>3000 mg/d	>600
Vitamin B12	750 µg	750 µg	750 µg	1500 µg	No adverse effects of 100 mg/d	>100

Recruitment of participants for the in-depth study.
As described above, schools will be randomly selected from a list of all eligible schools in Kampung Speu province. Once schools have been selected, schools will be informed of the

study, and a complete list of names of the children attending the school will be requested, as well as their class and gender. From this list, 150 children per school will be randomly selected, stratified for gender and class. As we expect that ~ 10% of the children will not be available (e.g. moved out of the area over the summer holidays), for each school we will generate a list of 180 children (20% excess). The school monitor will visit all homes of the children in the month before the school starts to obtain written informed consent of the parent for the in-depth study, and the first 150 children who will be present after the school holidays and whose parents agree to the study will be selected from each school.

Ethical consent of all participants.
During the first school week, information meetings will be organized for parents of all children, in which parents will be informed of the study, and that data on absenteeism and morbidity of their children will be recorded during the whole year. All parents will be asked for a signed informed consent form. Data will not be collected from children whose parents do not want to participate in the study.
(please see attached informed consent forms: 1x in-depth and 1x general)

Data collection and usage
Data on school attendance and reason for absence and morbidity will be collected from all children in the participating schools on a daily basis. This data will be used to assess the impact of the school meal program and the introduction of fortified rice on days absent and general health. Anthropometrical and cognitive data and biological samples will be collected in the sub-group of children participating in the in-depth study (25% of all children) at baseline (T0), after 5 months (T5, mid-way) and at the end of the school year after 10 months (T10), with the exception of stool samples, as these will be collected more frequently to assess the intestinal parasite re-infection rate. Children for the in-depth study will be selected at random from each school, using a computer list with all children attending the school, and specific informed consent will be obtained from the parents. For the in-depth study, age range will be limited to 8 – 13 years, due to the difficulties of obtaining reliable cognitive function tests in younger children, and the limited number of children with an age>13 still in primary school.
Anthropometrical data will be used to assess the impact of the school meal program (control) and the introduction of fortified rice (2 intervention groups) on the weight and height gain, and the acquisition of lean body mass (muscle mass). The cognitive data will be used to measure cognitive performance in the children, and to test whether improvements in micronutrient status are accompanied by increases in the ability to develop new insights and information from what is already perceived or known. The biochemical samples will be used to determine micronutrient and immune status (blood and urine samples) and parasite infestation and gut flora (stool sample).

For the implementation of the effectiveness study, monitors will be hired for the whole school year. For each school, 1 monitor will be hired (hence 20 in total). Before the start of the effectiveness study, school monitors will trained in a) providing information of the

study and answering questions related to the study, to vitamins and minerals in general and fortified rice; b) monitoring daily school attendance (using a prepared form with all children per class); c) monitoring reason of absence of children by home visit (scheduled for each afternoon after school finishes at noon). In addition to this, there will be 1 overall field coordinator, based in Kampung Speu province, to deal with day-to-day problems and questions.

For the collection of data (anthropometry, cognition) and samples (blood, urine, stool) teams will be formed for anthropometrical assessment, for cognitive function assessment and for biological sample collection. For anthropometry, it is estimated that 1 team of 4 persons can measure 50 school children in 1 morning. Therefore, 6 teams will be formed, to be able to measure 2500 children within 15 days

For cognitive function, it is estimated that 1 person will need 30 minutes to test 1 child. Therefore, to make sure that the cognitive team can also assess 50 children / school / morning, 1 team will consist of 8 persons will perform the cognitive assessment, with 6 teams for cognitive assessment in total. For the collection of biological samples, it is estimated that 1 team can obtain samples from 40 school children / day. Hence, 6 teams (of 3 persons) will be formed, to be able to measure 2500 children within 15 days.

Gaining consent

Written informed consent will be obtained from the parents or caretaker of each child participating in the study, as the study will be collecting data on morbidity and absence. In addition, a more elaborate informed consent will be obtained from the parents of caretakers of the children participating in the in-depth study. (See appendix)

Sample size considerations.

Reasons for collection of in-depth data from only a sub-group of children are that biochemical analysis of blood samples is expensive, and the cognitive tests are time consuming. However, as the biochemical analyses are sensitive to changes, a sample size of 500 children per group, is enough to show a significant effect of the fortified rice on hemoglobin concentration if the increase in hemoglobin concentrations is >4 g/L (average hemoglobin concentration 110 g/L). Moreover, the study can show a decrease in the prevalence of anemia of 9% or more (expected prevalence of anemia = 50%). For the other main biochemical indicators, a sample size of 500 children / group is sufficient to show biological significant impact. For objectives 5 (school attendance) and 7 (morbidity), a larger sample size is required to allow meaningful conclusions. Therefore, these outcomes will be followed in all 10000 children.

Tabulation of sample size calculations

**INDICATOR**		**EARLIER STUDIES**			**Sample size calculation for biochemical indicators**			
**Vitamin A status** (μmol/l)								
Mean	Expected effect	SD	α / β	Sample size	Desig n	Reference + comments	Current study	comments

					effect			
0.91	1.14	0.17	0.05 / 0.80	27	2	[25] Intake of 7 -12 g salt/d equals 1400 – 2400 IU Retinol/d. Effect after 1 yr of intervention	54	Design, which allows combining of groups if there are no significant interactions, not taken into account. Expected effect is at least 0.10 µmol/L
0.80	0.90	0.20	0.05 / 0.95	107	2		214	
1.00	1.05	0.20	0.05 / 0.80	270	2		540	
➔ Impact on vitamin A status is dependent on initial status, as plasma concentrations of vitamin A are under homeostatic control. Therefore, changes in plasma concentration are small when population mean is above 1.05 µmol/L								
**Iron status**								
**Hemoglobin (g/L)**								
116	129	12	0.05 / 0.80	42	2	[25] Intake of 7 -12 g salt/d 14 – 24 mg iron/d. Effect after 1 yr of intervention	84	
131	132	8.0	0.05 / 0.80	1050	2	[26] Fortified drink 1 yr	2100	This equals no impact
➔ Large differences in change in hemoglobin concentrations have been reported. Therefore, it is difficult to estimate a required sample size. Infection appears to be a major confounder on whether iron is used for hemoglobin production (no infection) or stored (infection)								
Serum ferritin								
15	31	30	0.05 / 0.80	168	2	[25] Intake of 7 -12 g salt/d 14 – 24 mg iron/d. Effect after 1 yr of intervention	336	
23	30	13	0.05 / 0.80	65	2	[26] Fortified drink 1 yr	130	
➔ A sample size of 500 children / arm is sufficient to detect physiological changes in iron storage (ferritin concentrations)								
Soluble Transferrin recept								
7.5	5.8	3.4	0.05 / 0.80	189	2	[25] Intake of 7 -12 g salt/d 14 – 24 mg iron/d. Effect after 1 yr of intervention	378	
➔ As for hemoglobin, large differences have been reported. However, a sample size of 500 children / arm is sufficient to detect meaningful changes in iron needs.								

Anthropometry

Prior to the study, all staff measuring anthropometry will be trained in the correct procedures for obtaining weight, height, arm circumference and skinfolds measurements. Staff will be re-trained before the mid-term and end-point data collection weeks. Weight will be measured on each child wearing light clothing using the Body Composition Monitor

Scale from Tanita BC-543, Japan to the nearest 0.1 kg. The accuracy of the scales will be checked every day using a set of calibration weights. Height of the children will be measured by a portable measuring tape (USA) to the nearest 0.1 cm. Height will be measured twice and the average value will be taken. If the two measures vary by more than 3 mm, a new set of two measures is taken until difference between the two is less than 3 mm. Arm circumference measurements (MUAC) will be made using a flexible, non-stretch tape. The measurement is taken at the midpoint of the upper left arm, between the acromion process and the tip of the olecranon. Triceps skinfold will be measured at the midpoint of the back of the upper left arm. All anthropometrical data will be recorded on a specific form which will be checked by the team supervisor daily.

Blood sampling

Blood sampling will be done at the village health centre. From a selected school, children (10/hr) will be brought by car to the village health centre, where a blood, urine and stool sample will be collected by the village health centre staff. Five (5) mL of venous blood will be drawn by venipuncture from the antecubital vein in a trace-element free vacutainer with anticoagulant (Vacuette, Greiner Bio One) by an experienced nurse from the local Health Centre in each village, using standard, sterile procedures. Immediately, 100 µl of whole blood will be used to fill a microcuvette for the determination of hemoglobin concentrations using the HemoCue system (HemoCue Angholm, Sweden). Certified control material will be measured daily prior to starting the measurements. All values of controls and duplicates will be noted and reported. Children with severe anemia (hemoglobin concentration < 70 g/L) will be send to the Provincial Health Centre for further diagnosis and treatment. The costs for transport, diagnosis and treatment will be for the project. The HemoCue Hemoglobin system consists of disposable microcuvettes with reagents in dry form and a single purpose designed photometer. No dilution is required. The photometer is calibrated at the factory against the cyanmethemoglobin method, which is the international reference method for the determination of the total hemoglobin concentration in blood. The remaining blood sample will be stored immediately in the dark in a cool box (4 C), and transported to the laboratory of Provincial Health Center (PHC) within 4h of blood collection. At the Provincial Health Center, whole blood will be centrifuged at 3000g for 10 min at room temperature and the plasma will be divided into five aliquots of 500 µl. Four aliquots of 500 µL will be put into pre-labeled eppendorf tubes and be frozen immediately at -20 C until transported to the laboratory of the National Institute of Public Health (NIPH) in Phnom Penh, where the samples will be frozen at -70°C until analysis. The last aliquot of 500 µl will be put into a 2.5 ml tube containing buffer (RPMI) and stimulant (LPS+PHA), and be incubated for 24 h at 37°C. Thereafter, the tube will be centrifuged and the supernatant collected and stored for -20C until transported to NIPH where after it will be stored at -70 C until analysis of cytokines produced.

Biochemical indicators of micronutrient and immune status

Iron status.

Iron status will be determined using plasma concentrations of 2 proteins: ferritin and soluble transferrin receptor (sTfR). Ferritin concentrations are a measure for the amount of

iron stored in the body. Concentrations <15 µg/L indicate depleted iron stores. sTfR concentrations are a measure for the demand of iron by the tissues. If iron becomes depleted in the tissue, sTfR concentrations will increase. Using a algorithm total iron body stores can be calculated. Both ferritin and sTfR plasma concentrations will be determined with commercially available ELISA kits (Ramco Inc). Plasma ferritin and transferrin receptor (aliquot 1) will be measured by ELISA using commercial kits (RAMCO) that include reference material, and will be performed at the Laboratory of NIPH. A 20 µL aliquot plasma will be used for the both the ferritin and TfR assay using the ELISA procedure (RAMCO Laboratories, Inc, Houston, Texas). The accuracy will be checked using WHO international standards (Ramco Laboratories).

Vitamin A status.
Vitamin A status is determined by measuring plasma retinol concentrations using a High Performance Liquid Chromatography with UV detection at 325 nm. *Plasma retinol* concentrations (aliquot 2) will be determined by HPLC (LC 10ADvp, Shimadzu, Japan,) according to the method of the International Vitamin A Consultative Group at the laboratory of the National Institute of Nutrition (NIN), Ministry of Health, Hanoi, Vietnam, using 400 µl of plasma and retinyl acetate as internal standard.

Zinc status.
Although plasma zinc concentrations are not strongly related to individual zinc status, due to the fact that many factors can affect zinc concentrations, plasma zinc concentrations are the best indicator for zinc status on a population level. *Plasma zinc* concentrations (aliquot 3) will be determined by flame atomic absorption spectrophotometry (spectrophotometer GBC, Avanta) using trace element-free procedures at NIN, Hanoi, Vietnam (2x 200 µl of plasma). Results will be verified against reference materials for zinc. To control for any potential contamination of material used for blood sampling and processing by external zinc, analysis will be carried out of the zinc content of at least 20 sets of material (needle, syringe, vacutainers and eppendorf tubes) when blood will be replaced by twice-distilled, deionized and demineralized water.

Inflammatory and immune status.
C-reactive protein (CRP) and α-1 acid-glycoprotein (AGP) are 2 acute phase proteins which rise early (CRP) and late (AGP) during an infection, under the influence of cytokines such as interleukin-6. ***CRP*** and ***AGP*** (aliquot 4), are measured to quantify the acute phase response to be able to correct for the effect of inflammation on the indicators described above. CRP, AGP and cytokine concentrations will be determined using commercial avaible ELISA kits (ICL Laboratories) which includes reference samples. 100 µL aliquots of plasma will be used for CRP and AGP assay, with 10% of the samples being done in duplicate. Assays will be done at the Laboratory of NIPH, Phnom Penh, Cambodia. ***Cytokines (IFN-y, IL-2, IL-6, IL-10, aliquot 5)*** will be measured as indicators of immune activation using commercially available ELISA kits (ICL Laboratories) at the laboratory of NIPH. Cut-offs for acute and chronic inflammation are a CRP>mg/L and a AGP>1.0 g/L respectively.

Iodine status.
Urinary iodine concentration is a reliable measure for iodine intake of an individual over

the last few days. The method used includes digestion of the urine sample with ammonium persulfate, a Sandell-Kolthoff reaction and reading at 340 nm. ***Urinary iodine concentrations*** will be measured at the National Institute of Nutrition in Hanoi, Vietnam.

Parasite infestation.

Parasites eggs (ascaris, trichuris, hook worm) will be counted in stool samples. Stool sample will stained and counted within one hour after staining by Kato-Katz method (WHO, 1994). The egg output will be expressed as mean eggs per gram faeces (epg).

Gut flora

Gut flora will be determined using bacterial DNA obtained from the stool samples with a commercial available kit for DNA extraction, using PCR techniques. Gut flora will be determined at the Free University Hospital, Amsterdam, The Netherlands.

Cognitive Function

Raven's Colored Progressive Matrices test will be used to measure the cognitive performance in the children. The test measures the ability to develop new insights and information from what is already perceived or known. The test uses 36 pictures with a pattern, in which children need to identify the missing piece out of a choice of 6 different options. The patterns increase in difficulty. The test is almost free of cultural bias as the test minimizes the impact of language skills and cultural bias and is appropriate for both children and adults. Raven's Matrices measure two complementary components of general intelligence: the capacity to think clearly and make sense of complex data (educative ability) and the capacity to store and reproduce information (reproductive ability). The Colored Matrices are especially suitable for children aged 5 to 11 years. Children will be tested individually by 1 team member of the cognitive team, while seated on a desk. In a separate room, 8 desks and chairs will be positioned with adequate space between them as not to have interference between the children while doing the testing. In addition, a series of cognitive tests will be selected from Wechsler's Intelligence Scale for Children III (WISC III), namely Digit span backward and forward, coding, and block design. The Digit span backward test assesses working memory for auditory information whereas the Digit span forward assesses children's auditory attention span and the ability to focus on auditory information. Coding requires children to quickly pair either shapes or numbers with a symbol and measures the speed of information processing. Block design is a measure of problem solving to assess executive function short-term memory and attention span. These tests were selected based on their wide usage, free of cultural bias and validity. All of these tests have been used successfully in earlier studies in Vietnamese schoolchildren.

The cognitive tests will be conducted by teams of university students who have been trained on the test methods and use of published materials translated into Khmer from the original test. The children will be assigned randomly to the technician. As for the other members of the research team, nobody in the team will be aware of the allocation of the school to control or fortified rice. However, we cannot guarantee that information concerning whether a school meal is served in the school or not (double-control) will be known to the field team. The duration of the whole range of tests is estimated to about 20-30 minutes. Each child will conduct the Raven's Colored Progressive Matrices test first,

followed by the other sub-tests of WISC III. Similar procedures will be conducted at baseline and endpoint survey. Because the test has not been standardized locally, interpretations of the scores of cognitive test will be used as raw scores.

Other measurements

In addition to the data collected from the school children, samples from the rice served in each school will be taken at baseline (after the start of the intervention), and every 2 months thereafter. Samples will therefore be taken at t=0, t=2, t=4, t=6, t=8 and t=10. In each school (n=16), 5 samples will be taken from the rice served in the classroom (from 5 different classes). Hence for each time point, 80 samples will be available, and for the whole duration of the intervention 480 samples will be available. These samples will be analyzed for micronutrient content (vitamin A, iron, zinc) at the Institute of Nutrition, Mahidol University, Bangkok, Thailand.

Data management and statistical analyses

A database will be constructed, using double entry of data to improve quality. All original data forms will contain the name of the participant to be able to check for the correct identity during data collection. The database however will be anonymized, with all participants being allocated a number which cannot be traced back to the participant without having the original data form. Original data forms will be stored at the Department of Fishery for up to 10 years after completion of the FORISCA project, and will then be destroyed.

Ethical considerations

Subjects of this intervention study will be schoolchildren from Grade 1 to Grade 6 (between 6 and 13 yrs of age). For the overall study, parents will be informed that their children might or might not receive rice during the whole school year which added vitamins and minerals. The amount of vitamins and minerals added will not exceed 50% of the recommended daily allowance. In addition, data on school attendance and morbidity will be kept. Parents can refuse either to data collection or to participate in the whole study. If parents refuse to have data collected on the attendance and morbidity of their child, the name of the child shall be omitted from the class list, and no information will be recorded. If parents refuse to participate in the study, a separate breakfast meal will be provided for the child. If many parents in a school refuse to participate (>5% of the parents), another school will be selected.

For the in-depth study, specific and detailed informed consent will be obtained. After selection, participants (parents of children and children) will be informed about the purposes and procedures of the study. At least one parent will be asked to sign the consent form presented in the annex. All refusals will be accepted without asking for reason of refusal. All respondents will be free to leave the survey at any time during the study, and this will be explained to the parents also. Before the implementation of the study, extensive consultation will be undertaken with the villages, the districts, and provincial authorities as well with school staff and parents of children. The protocol of the study will be submitted to

the Ethical Committee of the Ministry of Health, Kingdom of Cambodia, for approval before implementing the study.
Children with severe anemia (Hb < 70 g/L) will be referred to the Provincial Health centre for further diagnosis and treatment. All costs (diagnosis, transport and treatment) will be for the project. Children with mild anemia will not be treated, as the consequences of mild anemia on health are currently unclear.

Possible adverse effects.
The study will collect blood samples using vena-puncture. Besides being painful, possible adverse effects include collapse of the child and phlebitis or other infection at the site of blood drawing. Concerning collapse of the child, this is caused by a strong vaso-vagal nervous reaction, and although harmless in itself, can cause harm if the child is standing. Therefore, children will be seated in a comfortable chair during blood taking. The risk for phlebitis or other infections will be minimized by using sterile techniques and cleaning the area of the blood drawing with 70% alcohol before the actual blood draw.
No other possible adverse effects are foreseen.

Data monitoring board.
A data monitoring board will be formed who will review the data after the mid-term (T5) evaluation. Although data on morbidity might not be completely available at that time, data on hemoglobin and anemia prevalence will be available. Given the low incidence of mortality in school-aged children, we expect that there will be no data analysis on mortality. Mortality data analysis will be done if there are more than 5 cases.

References

1. WHO: Worldwide prevalence of anaemia 1993–2005 : WHO global database on anaemia. Edited by de Benoist BM, E.
Egli, I.
Cogswell, M. Geneva, World Health Organization, 2008, p.
2. WHO: Iron Deficiency Anaemia Assessment, Prevention and Control. A guide for programme managers. Edited by Geneva, World Health Organization, 2001, p.
3. Bhaskaram P: Micronutrient malnutrition, infection, and immunity: an overview, Nutr Rev 2002, 60:S40-45
4. WHO: Global prevalence of vitamin A deficiency in populations at risk 1995–2005: WHO global database on
vitamin A deficiency. Edited by 2009, p.
5. Dijkhuizen MA, Wieringa FT, West CE, Muherdiyantiningsih, Muhilal: Concurrent micronutrient deficiencies in lactating mothers and their infants in Indonesia., Am J Clin Nutr 2001, 73:786-791
6. Dijkhuizen MA, Wieringa FT, West CE, Muhilal: Zinc plus beta-carotene supplementation of pregnant women is superior to beta-carotene supplementation alone in improving vitamin A status in both mothers and infants, Am J Clin Nutr 2004, 80:1299-1307
7. Suharno D, West CE, Muhilal, Karyadi D, Hautvast JG: Supplementation with vitamin A and iron for nutritional anaemia in pregnant women in West Java, Indonesia, Lancet 1993, 342:1325-1328
8. Zimmermann MB, Biebinger R, Rohner F, Dib A, Zeder C, Hurrell RF, Chaouki N: Vitamin A supplementation in children with poor vitamin A and iron status increases erythropoietin and hemoglobin concentrations without changing total body iron, Am J Clin Nutr 2006, 84:580-586
9. Biebinger R, Arnold M, Koss M, Kloeckener-Gruissem B, Langhans W, Hurrell RF, Zimmermann MB: Effect of concurrent vitamin A and iodine deficiencies on the thyroid-pituitary axis in rats, Thyroid 2006, 16:961-965
10. Allen LH: Nutritional influences on linear growth: a general review. , Eur J Clin Nutr 1994, 48 Suppl 1:S75-S89
11. Black MM: Micronutrient deficiencies and cognitive functioning, J Nutr 2003, 133:3927S-3931S
12. Qian M, Wang D, Watkins WE, Gebski V, Yan YQ, Li M, Chen ZP: The effects of iodine on intelligence in children: a meta-analysis of studies conducted in China, Asia Pac J Clin Nutr 2005, 14:32-42
13. Pollitt E, Jahari A, Husaini M, Kariger P, Saco-Pollitt C: Developmental trajectories of poorly nourished toddlers that received a micronutrient supplement with and without energy, Journal of Nutrition 2002, 132:2617-2625
14. Black MM, Baqui AH, Zaman K, Ake Persson L, El Arifeen S, Le K, McNary SW, Parveen M, Hamadani JD, Black RE: Iron and zinc supplementation promote motor development and exploratory behavior among Bangladeshi infants, Am J Clin Nutr 2004, 80:903-910

15. McCann JC, Ames BN: An overview of evidence for a causal relation between iron deficiency during development and deficits in cognitive or behavioral function, Am J Clin Nutr 2007, 85:931-945
16. Black MM, Sazawal S, Black RE, Khosla S, Kumar J, Menon V: Cognitive and motor development among small-for-gestational-age infants: impact of zinc supplementation, birth weight, and caregiving practices, Pediatrics 2004, 113:1297-1305
17. Hall A: Micronutrient supplements for children after deworming, Lancet Infect Dis 2007, 7:297-302
18. Hall A, Hewitt G, Tuffrey V, de Silva N: A review and meta-analysis of the impact of intestinal worms on child growth and nutrition, Matern Child Nutr 2008, 4 Suppl 1:118-236
19. Lee KJ, Bae YT, Kim DH, Deung YK, Ryang YS, Kim HJ, Im KI, Yong TS: Status of intestinal parasites infection among primary school children in Kampongcham, Cambodia, Korean J Parasitol 2002, 40:153-155
20. Nga TT, Winichagoon P, Dijkhuizen MA, Khan NC, Wasantwisut E, Wieringa FT: Decreased parasite load and improved cognitive outcomes caused by deworming and consumption of multi-micronutrient fortified biscuits in rural vietnamese schoolchildren, Am J Trop Med Hyg 2011, 85:333-340
21. Black RE, Allen LH, Bhutta ZA, Caulfield LE, de Onis M, Ezzati M, Mathers C, Rivera J: Maternal and child undernutrition: global and regional exposures and health consequences, Lancet 2008, 371:243-260
22. WFP: Cambodia School Feeding Impact Evaluation. Edited by Evaluation Oo. Rome, 2011, p.
23. Horton S: The economics of food fortification, J Nutr 2006, 136:1068-1071
24. GAIN: Grain Industry in Cambodia. (http://www.fas.usda.gov/gainfiles/200603/146187256.pdf). Edited by USDA Foreign Agriculture Service. , 2006, p.
25. Zimmermann MB, Wegmueller R, Zeder C, Chaouki N, Biebinger R, Hurrell RF, Windhab E: Triple fortification of salt with microcapsules of iodine, iron, and vitamin A, Am J Clin Nutr 2004, 80:1283-1290
26. Osendarp SJ, Baghurst KI, Bryan J, Calvaresi E, Hughes D, Hussaini M, Karyadi SJ, van Klinken BJ, van der Knaap HC, Lukito W, Mikarsa W, Transler C, Wilson C: Effect of a 12-mo micronutrient intervention on learning and memory in well-nourished and marginally nourished school-aged children: 2 parallel, randomized, placebo-controlled studies in Australia and Indonesia, Am J Clin Nutr 2007, 86:1082-1093
27. Gibson RS: Principles of nutritional assessment. Edited by Oxford, Oxford University Press, 1990, p
28. Zimmermann MB: Methods to assess iron and iodine status, Br J Nutr 2008, 99 Suppl 3:S2-9
29. WHO: Edited by Geneva, World Health Organization, 1996, p. pp. 72-104
30. Assessment of the risk of zinc deficiency in populations and options for its control. IZINCG technical document #1. Edited by Brown KH, Hotz,C. International Nutrition Foundation for United Nations University Press, 2004, p

31. Wieringa FT, Dijkhuizen MA, West CE, Northrop-Clewes CA, Muhilal: Estimation of the effect of the acute phase response on indicators of micronutrient status in Indonesian infants., Journal of Nutrition 2002, 132:3061-3066

32. Nga TT, Winichagoon P, Dijkhuizen MA, Khan NC, Wasantwisut E, Furr H, Wieringa FT: Multi-micronutrient-fortified biscuits decreased prevalence of anemia and improved micronutrient status and effectiveness of deworming in rural Vietnamese school children, J Nutr 2009, 139:1013-1021

ANNEX 3 – TIME SCHEDULE

2012 2012 | 2013

2013

MAY	JUNE	JULY	AUG	SEPT	OCT	NO	DEC	JAN	FEB	MAR	APRIL	MAY	JUNE	JULY	AUG	SEPT	OCT	NOV	DEC
PREPARE DATA COLLECTION																			
	SELECT + RANDOMIZE SCHOOLS																		
		PREPARE LIST OF CHILDREN																	
			RANDOMIZE CHILDREN																
			TRAIN ANTHROP + COGNITION TEAMS																
				TRAIN BLOOD + URINE TEAMS															
					BASELINE DATA COLLECTION														
						S													
						T													
						O			MID-POINT DATA COLLECTION										
													END-POINT DATA COLLECTION						
												SAMPLE ANALYSIS							

														**DATA ANALYSIS AND REPORTING**

APPENDIX 4 – ANTHROPOMETRY FORMS

ការសិក្សាមនុស្សមាត្រវិទ្យា

Anthropometry

The FORISCA PROJECT

Village: ___________________ School name:_____________________________ School number: _______

Date of data collection _____ / _____ / ______

Baseline / Mid-point (5 months) / Endline (10 months)

---

1. *Anthropometry*

1.1 *Weight (record two measurements):*	
weight1 ____ . __ kg	1.1.2 weight2 ____ . __ kg
1.2 *Length (record two measurements)*	
Length1 _______ . __ cm	Length2 _______ . __ cm
1.3 MUAC *(record two measurements)*	
MUAC1 ___ . __ cm	MUAC2 ____ . __ cm
1.4 *Triceps Skinfold Thickness (TSF) (record two measurements)*	
TSF1 ____ . __ mm	TSF2 ____ . __ mm
1.5 *Subscapular Skinfold Thickness (SSF) (record two measurements)*	
SSF1 ____ . __ mm	SSF2 ____ . __ mm

Any Comments:

....

....

គ្រប់សំនួរទាំងអស់ត្រូវបានផ្ទៀងផ្ទាត់ឡើងវិញដោយ: ______________________________

(All answers have been cross-checked by)

Annex 5 --- Ethical Consent Form

INFORMATION FOR PARTICIPANTS IN A STUDY TITLED:

Introduction of Fortified Rice for School Meals in Cambodia (FORISCA) to Improve Health and Development of Cambodian School Children

Hello, my name is _*(interviewer's name)*_____. I work with the Institute of Fisheries Post-Harvest Technologies, Fishery administration of the Ministry of Agriculture, Cambodia. I would like to inform you on a study we are planning to do at the school of your child in the coming year, and ask for your approval for your child to participate in this study.

School children are vulnerable for a lack of good nutrition (undernutrition) which can lead to a lack of vitamins and minerals (micronutrient deficiencies). Children need vitamins and minerals to stay healthy and to be able to do well at school. We are doing a study, in which we will compare three types of rice that have added vitamins and minerals. Most children in the study (in Kampung Speu) will get rice through the normal school meal program from the World Food Program school meal project.

- If the school of your child is in the program from WFP, this program will continue as normal. However, in some schools, we will replace the normal rice with the fortified rice, that is, the rice with the extra vitamins and minerals.
- If the school of your child is not participating in the program of WFP, also nothing will change. For the coming year, the school will not be included, and there will be no school meal served at school.

Questionnaires

We want to know the benefits of the fortified rice on the health and learning capabilities of the school children. Therefore, we would like to follow the children in the schools for 1 whole school year (from October 2012 until July 2013). We will record the names of the children who are absent each day, and we will come to your house to ask why the child was not at school. Maybe because the child was ill, or maybe for some other reason. We will not give this information to school, but record it only for the study, and it has no consequences for the school meal your child is receiving nor for anything else. If the child was ill, we would like to know what kind of symptoms the child had.

In-depth study

In addition to the questions asked above, we would like to have more detailed information on the nutritional status and learning capacities of the children. For this, we will need to collect blood, stool and urine samples. However, we will not collect these data in all children, only in ¼ of the children in the school. Your child has been selected to be one of those to give blood, urine and stool samples. Blood and urine samples will be collected 3 times over the year (as baseline, after 5 months and at the end of the school year). Stool samples will be collected every 2 months.

The amount of blood taken will be 5 ml, or about one teaspoon of blood from your child's inner elbow. We will test your child's blood for anemia, that is, whether s/he has enough blood cells, and for vitamin and minerals in the blood (vitamin A, iron and zinc, B-vitamins, vitamin D), general infection and inflammation. The blood will not be used for anything else. If we find your child has severe anemia, we will refer him/her to the Health Centre for treatment. All costs for this will be carried by the project, and will not be yours. The stool and urine sample will be used to look for iodine deficiency and for parasites.

Your participation is entirely your choice. Whether you choose to participate or not, will not affect the school meal your child might be receiving, nor anything else. Although we hope you will continue with the study for the full 10 month period (October 2012 – July 2013), you can stop participating with the study any time during the study, and only have to tell you that you don't want your child to participate anymore in the study.

If at any time during the study you have any questions, you can call this number –

(INCLUDE PHONE NUMBER OF STAFF RESPONSIBLE)

We would like to ask for your participation in the study now. If you agree to participate with your child in the study, please sign or mark your mark in the box below.

Signature or thumbprint of the caregiver:

For study fieldworker

I have read the consent form in its entirety to the caregiver of the child.

Signature of study fieldworker:

____

Date (day/month/year):

_______/_______/________

Leave a copy of the consent form with the caregiver. Circle the telephone number on the page to ensure that they understand they can call for more information.

ANNEX 6 – ETHICAL ISSUES.

Ethical considerations

Subjects of this intervention study will be schoolchildren from Grade 1 to Grade 6 (between 6 and 13 yrs of age). For the overall study, parents will be informed that their children might or might not receive rice during the whole school year which added vitamins and minerals. The amount of vitamins and minerals added will not exceed 50% of the recommended daily allowance. In addition, data on school attendance and morbidity will be kept. Parents can refuse either to data collection or to participate in the whole study. If parents refuse to have data collected on the attendance and morbidity of their child, the name of the child shall be omitted from the class list, and no information will be recorded. If parents refuse to participate in the study, a separate breakfast meal will be provided for the child. If many parents in a school refuse to participate (>5% of the parents), another school will be selected.

For the in-depth study, specific and detailed informed consent will be obtained. After selection, participants (parents of children and children) will be informed about the purposes and procedures of the study. At least one parent will be asked to sign the consent form presented in the annex. All refusals will be accepted without asking for reason of refusal. All respondents will be free to leave the survey at any time during the study, and this will be explained to the parents also. Before the implementation of the study, extensive consultation will be undertaken with the villages, the districts, and provincial authorities as well with school staff and parents of children. The protocol of the study will be submitted to the Ethical Committee of the Ministry of Health, Kingdom of Cambodia, for approval before implementing the study.

Children with severe anemia (Hb < 70 g/L) will be referred to the Provincial Health centre for further diagnosis and treatment. All costs (diagnosis, transport and treatment) will be for the project. Children with mild anemia will not be treated, as the consequences of mild anemia on health are currently unclear.

Possible adverse effects.

The study will collect blood samples using vena-puncture. Besides being painful, possible adverse effects include collapse of the child and phlebitis or other infection at the site of blood drawing. Concerning collapse of the child, this is caused by a strong vaso-vagal nervous reaction, and although harmless in itself, can cause harm if the child is standing. Therefore, children will be seated in a comfortable chair during blood taking. The risk for phlebitis or other infections will be minimized by using sterile techniques and cleaning the area of the blood drawing with 70% alcohol before the actual blood draw.

No other possible adverse effects are foreseen.

The collection of urine and stool samples does not pose any health risk, although it might give an uncomfortable feeling for some children and/or their parents.

Annex 7 – COMPENSATION MECHANISMS

There will be no financial compensation for any of the children and/or their parents just for participating in the study. All children will receive a school meal, 6 days/week as intended by the WFP's school meal program, or no school meal, when the school they are visiting is not participating in the WFP school meal program. However, medical costs and transport costs will be paid by the project for any child being diagnosed with a hemoglobin concentration of less than 70 g/L (severe anemia). It is expected that ~5% of the children will have severe anemia, for which appropriate medical treatment will be given.

Annex 8 – Curriculum Vitae

Name: WIERINGA, Frank Tammo
Nationality: Dutch.
Birth; date, place: 5 September 1969, Naarden, The Netherlands.
Languages: Dutch (excellent), English (excellent), French (good), Bahasa Indonesia (good), German (fair).
Education:
- M.Sc. Chemistry, Specialization Biochemistry, University of Amsterdam, 1993.
- M.Sc. Medicine, University of Amsterdam, 1995.
- Ph.D. in nutritional sciences, University of Wageningen, Netherlands, 2001
- M.D. University of Amsterdam, September 2003.

Professional experience:
- September 1995 – December 1995. Medical officer, Kaoma District Hospital Zambia.
- 1996 – 2001. PhD fellow, based at Nutrition Research Centre, Bogor, Indonesia
- November 2003. Consultant for UNICEF at Inst. Nutrition, Mahidol Univ. Bangkok.
- March 2004 - December 2007. Post-doctoral fellow at the Dept. Intern. Med. University Medical Center Nijmegen, The Netherlands.
- December 2007 – present. Senior Reseacher at IRD, Montpellier, France, based at NIN, Hanoi, Vietnam.

Research projects currently on-going (January 2012):
- Micronutrient supplementation in low-birth-weight Vietnamese infants. Effects on micronutrient status and growth. PhD project Phan Bich Nga, NIN
- Determining the role of additional vitamins and minerals, next to ORS and zinc, in the recovery from diarrhea in Vietnamese children. PhD project Thi My Thuc, NIN
- Determining the role of the acute phase response on the effectiveness of post-partum vitamin A supplementation. PI: F.T. Wieringa, Post-doc: Tran Thuy Nga, NIN
- Development of a locally produced Ready-to-Use-Therapeutic-Food (RUTF) in Vietnam for prevention and treatment of malnutrition. Joint collaboration IRD & NIN
- Efficacy of a locally produced Ready-to-Use-Therapeutic-Food (RUTF) in Vietnam in the treatment of malnutrition. Joint collaboration IRD, NIN and UNICEF.
- Introducing Fortified Rice (UltraRice, PATH) in Vietnam. Identification of constraints and opportunities. PhD project Tran Khanh Van, NIN, Vietnam
- Development of a locally produced complementary food to improve micronutrient status and prevent growth faltering in young infants and children in Cambodia. Joint project Copenhagen University, IRD and WFP-Cambodia (WinFood). PhD project J. Skau, DK.
- First national micronutrient survey Vietnam. Joint project NIN, GAIN and IRD. PhD project Arnauld Laillou, GAIN.

Professional experience:
- September 1995 – December 1995. House officer at Kaoma District Hospital, Kaoma Zambia.
- 1996 – 2001 PhD fellow. Research project at the Nutrition Research and Development Centre, Ministry of Health, Bogor, Indonesia, Dept. of Human Nutrition and

Epidemiology, Wageningen University Netherlands, and Dept. Internal Medicine, Catholic University Nijmegen, Netherlands.
- November 1999. Consultant for UNOPS in Sulawesi, Indonesia. Assessment of nutritional impact of PUTKATI-project as part of external consultancy team for half-term interim project evaluation.
- November 2001 - September 2003. House officer at the Academic Medical Centre (Amsterdam) and various other hospitals affiliated to the University of Amsterdam.
- May 2003. Consultant in Bangladesh for Free University Amsterdam. Evaluation and project development of IPSAD project DGIS, Min. Foreign Affairs, Netherlands and Grameen Foundation, Bangladesh.
- November 2003 – December 2003. Consultant for UNICEF, based at Institute of Nutrition, Mahidol University (INMU), Bangkok, Thailand for the Multi-Country Trial of Iron and Zinc Supplementation in Infants. Compilation and analysis of pooled database, drafting of report and recommendations in collaboration with INMU.
- March 2004 - December 2007. Post-doctoral research fellow at the Dept. Int. Med., Univ. Medical Center Nijmegen, The Netherlands, on tuberculosis and wasting, based at Hasan Sadikin Hospital, University Padjadjaran, Bandung, Indonesia.
- December 2007 – onwards. Senior Reseacher at IRD, UMR 204, NutriPass: Prevention of Malnutrition and associated pathologies, Montpellier, France.

Teaching experience:
- 1997 – 2000. Lecturing, and supervising work-groups and practicals for the M.Sc. course in Human Nutrition, Wageningen University, The Netherlands.
- 1997 – 2001. Lecturing, and supervising work-groups and practicals for the M.Sc. course in Nutrition, SEAMEO-TROPMED, University of Indonesia, Jakarta.
- 1997 – 2001. Training of research and laboratory staff in quality control and the analysis of trace elements, vitamin A and carotenoids at the Nutrition Research and Development Centre, Ministry of Health, Bogor, Indonesia and SEAMEO-TROPMED, University of Indonesia, Jakarta, Indonesia.
- 2001 – 2003. Supervising work-groups on “Issues in Tropical Medicine”, Dept. of Social Medicine, University of Amsterdam, The Netherlands.
- 2003. Lecturing in the International Course in Nutrition, International Agricultural Center, Wageningen, The Netherlands on Iodine, Vitamin A, Iron and Zinc.
- 2003 – 2007. Lecturing at the Faculty of Science, Mahidol University, Bangkok, Thailand. Core lectures at the Institute of Nutrition, Mahidol University for M.Sc. and Ph.D program in Nutrition.
- 2005 – onwards. Guest lecturer at the Department of Human Nutrition, Copenhagen University, Denmark.
- 2008 – onwards. Lecturing at the National Institute of Nutrition, Hanoi, Vietnam

Research projects finalized:
- Gentamycin Pharmacokinetics in Malnourished Infants. Tropical Metabolism Research Unit, University of the West Indies, Jamaica. 1990-1991.
- Inflammatory Response in Malnourished Infants. Tropical Metabolism Research Unit, University of the West Indies, Jamaica. 1990-1991.

- Identification of biological active peptides (Magainins), and their effect on different cell membranes, Dutch Cancer Institute, University of Amsterdam (M.Sc. Research Thesis Biochemistry). 1991-1992.
- The nature of the scrapie agent. University of Amsterdam, June 1992 (M.Sc. Thesis Biochemistry).
- Development of an information system with health flags as a way of improving the quality of primary health care in rural Zambia. Kaoma District Hospital, Kaoma, Zambia. September 1995 - December 1995.
- Vitamin A, Iron and Zinc Deficiency in Indonesia. Micronutrient Interactions and Effects of Supplementation. Nutrition Research and Development Centre, Bogor, Indonesia, in collaboration with Wageningen University and Catholic University Nijmegen, The Netherlands. 1996-2001.
- Multi-micronutrient supplementation and deworming in Vietnamese schoolchildren. Interactions and effects on cognitive function. Collaboration between Mahidol University, Bangkok Thailand and National Institute of Nutrition, Hanoi, Vietnam. 2003-onwards.
- Tuberculosis and Wasting: the role of micronutrients in body composition changes during TB treatment. Post-doctoral research funded by the Dutch Foundation for the Advancement of Tropical Research (WOTRO), 2004-2008.
- Role of micro- and macronutrients in determining the body composition in Thai HIV patients. Research funded by the IAEA, Vienna. 2006-2009.

Selected scientific Publications:

- Doherty JF, Dijkhuizen MA, Wieringa FT, Moule N, Golden MHN. WHO definition of tachypnoea in children. Lancet(1991);338:1454.
- Dijkhuizen MA, Wieringa FT, West CE, Muherdiyantiningsih, Muhilal. Concurrent micronutrient deficiency in lactating mothers and their infants in Indonesia. Am.J.Clin.Nutr. (2001);73:786-91
- Dijkhuizen MA, Wieringa FT, West CE, Sri Martuti, Muhilal. Effects of Iron and Zinc Supplementation in Indonesian Infants on Micronutrient Status and Growth. J. Nutr. (2001);131:2860-5
- Wieringa FT, Dijkhuizen MA, West CE, Northrop-Clewes CA, Muhilal. Estimation of the effect of the acute phase response on indicators of micronutrient status in Indonesian infants. J.Nutr.(2002);132: 3061-3066.
- Wieringa FT, Dijkhuizen MA, West CE, Thurnham DI, Muhilal, Van der Meer JWM. Redistribution of vitamin A after iron supplementation in Indonesian infants. Am.J.Clin.Nutr.(2003);77:651-57.
- Dijkhuizen MA, Wieringa FT, West CE, Muhilal. Micronutrient deficiency and supplementation in Indonesian infants. Interactions among micronutrients. Adv.Exp.Med.Biol. (2003);531:359-68.
- Wieringa FT, Dijkhuizen MA, Van der Ven-Jongekrijg J, West CE, Muhilal, Van der Meer JWM. Micronutrient deficiency and supplementation in Indonesian infants. Effects on immune function. Adv.Exp.Med. Biol.(2003); 531:369-77.
- Dijkhuizen MA, Wieringa FT, West CE and Muhilal. Zinc plus β-carotene supplementation of pregnant women is superior to β-carotene alone in improving vitamin A status of mothers and infants. Am. J. Clin. Nutr. (2004);80:1299-307.
- Wieringa FT, Dijkhuizen MA, West CE, van der Ven-Jongekrijg J, van der Meer JW, Muhilal. Reduced production of immunoregulatory cytokines in vitamin A- and zinc-deficient Indonesian infants. Eur. J. Clin. Nutr. (2004);58:1498-504.
- Van Lettow M, West CE, van der Meer JW, Wieringa FT, Semba RD. Low plasma selenium concentrations, high plasma human immunodeficiency virus load and high interleukin-6 concentrations are risk factors associated with anemia in adults presenting with pulmonary tuberculosis in Zomba district, Malawi. Eur J Clin Nutr (2005);59:526-32.

- Wieringa FT, Berger J, Dijkhuizen MA, Hidayat A, Ninh NX, Utomo B, Wasantwisut E, Winichagoon P. Combined iron and zinc supplementation in infants improved iron and zinc status, but interactions reduced efficacy in a multicountry trial in southeast Asia. J Nutr (2007): 137:466-72.
- Wieringa FT, Berger J, Dijkhuizen MA, Hidayat A, Ninh NX, Utomo B, Wasantwisut E, Winichagoon P. Sex differences in prevalences of anemia and iron deficiency in infancy in a large multi-country trial in SE-Asia. Brit. J. of Nutr. (2007); 98:1070-1076
- Sahiratmadja E, Wieringa FT, van Crevel R, et al. Iron deficiency and NRAMP1 polymorphisms (INT4, D543N and 3/UTR) do not contribute to severity of anaemia in tuberculosis in the Indonesian population. Brit. J. of Nutr. 2007;98:684-690.
- Wieringa FT, Dijkhuizen MA, van der Meer JWM. Maternal micronutrient supplementation and child survival. Lancet 2008 (May 24);371(9626):1751-2
- Dijkhuizen MA, Winichagoon P, Wieringa FT, Wasantwisut E, Utomo B, Ninh NX, Hidayat A, Berger J. Zinc Supplementation Improved Length Growth Only in Anemic Infants in a Multi-Country Trial of Iron and Zinc Supplementation in South-East Asia. J. Nutr. (2008) 138: 1969–1975.
- Nga TT, Winichagoon P, Dijkhuizen MA, Khan NC, Wasantwisut E, Furr H, Wieringa FT. Multi-micronutrient-fortified biscuits decreased prevalence of anemia and improved micronutrient status and effectiveness of deworming in rural Vietnamese school children. J Nutr. 2009;139(5):1013-20.
- Friis H, Gomo E, Mashange W, Nyazema N, Kæstel P, Wieringa FT, Krarup H. The Acute Phase Response to Parturition: A Cross-Sectional Study in Zimbabwe. Afr J Reprod Health 2009; 13:61-68.
- Thurnham DI, McCabe LD, Haldar S, Wieringa FT, Northrop-Clewes CA, McCabe GP. Adjusting plasma ferritin concentrations to remove the effects of subclinical inflammation in the assessment of iron deficiency: a meta-analysis. Am J Clin Nutr. 2010 Sep;92(3):546-55.
- Wieringa FT, Dijkhuizen MA, Muhilal, Van der Meer JW. Maternal micronutrient supplementation with zinc and β-carotene affects morbidity and immune function of infants during the first 6 months of life. Eur J Clin Nutr. 2010 Oct;64(10):1072-9
- Phu PV, Hoan NV, Salvignol B, Treche S, Wieringa FT, Khan NC, Tuong PD, Berger J. Complementary Foods Fortified with Micronutrients Prevent Iron Deficiency and Anemia in Vietnamese infants. J Nutr. 2010 12: 2241-7
- Nga TT, Winichagoon P, Dijkhuizen MA, Khan NC, Wasantwisut E, Wieringa FT. Deworming and consumption of multi-micronutrient fortified biscuits decreased parasite load and improved some cognitive outcomes in rural vietnamese schoolchildren. Am J Trop Med Hyg 2011 Aug;85(2):333-40.
- Berger J, Wieringa FT, Lacroux A, Dijkhuizen MA (2011). Strategies to prevent iron deficiency and improve reproductive health: opportunities, efficacy, effectiveness and safety issues. Who to target and why, what works and what might work. Nutr Rev 2011:69 Suppl 1:S78-86.
- Wieringa FT, Dijkhuizen MA, Winichagoon P, Wasantwisut E, Utomo B, Ninh NX, Hidayat A, Berger J. Growth, Stunting, and Micronutrient Supplementation: Perspectives from the South-East Asia Multi-country Trial of Iron and Zinc Supplementation in Infants (SEAMTIZI) in: Handbook of Growth and Growth Monitoring in Health and Disease. Springer Verlag 2012: Chapter 39
- Hieu NT, Sandalinas F, de Sesmaisons A, Laillou A, Tam NP, Khan NC, Bruyeron O, Wieringa FT, Berger J. Multi-micronutrient-fortified biscuits decreased the prevalence of anaemia and improved iron status, whereas weekly iron supplementation only improved iron status in Vietnamese school children. Br J Nutr. 2012:1-9.
- Wieringa FT, Dijkhuizen MA, Berger J. Vitamin A supplementation in children and hearing loss. BMJ. 2012;344.

Sadaf Khan
2201 Westlake Ave, Suite 200
Seattle, WA 98121
Phone:206-302-4812
e mail: sakhan@path.org

PROFILE : Public health professional with a focus on reproductive health and maternal, neonatal and child health. Strong track record of developing, managing and evaluating field programs in maternal and child health internationally. Extensive experience collaborating with academics, officials from non-govermental and governmental organizations, community members and program managers.

EDUCATION:

Degree	Year	Institution	Grades
Doctor of Public Health (DrPH)	2010	Johns Hopkins Bloomberg School of Public Health, Baltimore MD, USA<br>**Dissertation title:** ***Maternal Health: A Study of Individual and Community Factors Related to Care-Seeking and Maternal Mortality In Pakistan***	GPA: 4.0
Master of Public Health (MPH)	2003	Johns Hopkins Bloomberg School of Public Health, Baltimore MD, USA	GPA 3.92
Bachelor of Medicine and Bachelor of Surgery (M.B.B.S)	1998	Dow Medical College Karachi	In the top 5% of a graduating class of 500

CONTINUING EDUCATION:

Participated in Workshops on:

- Population Policy Communications-as part of the Population Reference Bureau's (PRB) Fellows Program. Washington DC: June 2009.
- Methodological Issues in Health Research and Reproductive Health: Conducted by the Geneva Foundation of Medical Education and Research (GFMER) Geneva, Switzerland: February-March 2005.
- Sexuality & Sexual Health. Conducted by Aahung, Pakistan. January 2005.
- Leadership Training in Reproductive Health, organized by Population Council Pakistan. May-June 2004.

- Strategic Leadership for Population and Reproductive Health. Conducted by the Bill & Melinda Gates Institute for Population and Reproductive Health and Johns Hopkins University Bloomberg School of Public Health. January 2003
- Contraception and High Risk Pregnancies. Conducted by the Dept. of Obstetrics and Gynecology, Liaquat National Hospital, Karachi. June 2002
- Integrated Management of Childhood Illness, as part of the Pakistan Pediatric Association's Biennial Conference. February 2002
- Population and Reproductive Health. Organized by the Ministry of Population Welfare. June 2000
- The Lactation Management Program, Conducted by the Lactation Management Programme, Sindh, June 1999

EXPERIENCE:

<u>August 2010-Present :</u>

PATH

Senior Newborn, Maternal, and Child Health Specialist

Job responsibilities include:

- Providing technical guidance and support for a landscape analysis of maternal and perinatal infections in Bangladesh, India and Uganda.
- Providing technical leadership and support to country and project offices on maternal, newborn and child health activities.
- Leading and coordinating project activities planning for the introduction of Depo-SubQ in Uniject ® in Pakistan
- Provision of scientific leadership, oversight and management to international field trials and research involving human subjects
- Serving as a liaison with the Technology Solutions team around technologies for maternal and child health

<u>September 2005- May 2010:</u>

Johns Hopkins University Bloomberg School of Public Health

Graduate Research Assistant/Student Researcher, Department of Population, Family and Reproductive Health.

Research assistance and support on development of advocacy materials, research reports and preparation of scientific grants.

Projects included:

- *Making the Case for U.S. International Family Planning Assistance*
- Maternal health care and service provision in developing countries.
- Health benefits of contraception
- Factors associated with non-accidental violent deaths amongst women in Bangladesh
- Exploring adoption of maternal and child health technologies using Everett Roger's Diffusion of Innovation Theory

August 2003 to August 2005:

Ziauddin Medical University, Karachi.
Assistant Professor, Community Health Sciences (CHS)
Job responsibilities included:

- **Curricular and Teaching Experience**:

- Team leader of a group working to incorporate gender and women's health issues into the University's undergraduate medical curriculum. The project involved collaborating with senior professionals from basic sciences, medical and surgical specialties.
- Ran research methodology workshops for faculty and post-graduate students.
- Coordinated CHS curriculum of undergraduate medical students; developed and expanded the reproductive health curriculum.
- Worked on revision of the CHS curriculum.
- Involved in teaching modules of Epidemiology, Biostatistics, Reproductive Health, Maternal and Child Health and Public Health Nutrition to undergraduate medical students.

- **Research:** Planning and supervising research studies at the University's field sites. Facilitating students and other faculty members in their research projects. Designing and implementing census and sample surveys at the University's field sites.
- **Program Management and Evaluation**: Managing and coordinating primary health care activities of field sites (population ~25,000). Organized and took primary responsibility for managing center-based and outreach programs for maternal and child health and family planning. Lead researcher for an impact assessment of health service provision on Reproductive Health and Safe Motherhood indicators at the field site.
- **Health Education**: Developed outlines for individual and group counseling (center based as well as outreach) pertaining to reproductive health and child health in communities served by the University.
- **Capacity Building:** Initiated and managed a training program on management protocols for maternal and child health for Community Health Workers .
- **Consulting clinics**: Involving preventive and promotive health care, family planning, immunization, oral rehydration therapy and growth monitoring in addition to routine general consultations and antenatal care. Supervised a staff of six physicians and ten auxiliary health workers.

April 2000 to June 2002

Ziauddin Medical University, Karachi.
Instructor, Community Health Sciences (CHS)

June 1999 to December 1999:

Department of E.N.T, Civil Hospital Karachi
House Officer.

Job responsibilities included:

- In -patient care as well as consultations in the Out Patient Department
- Performing minor surgeries and assisting in major surgeries.

December 1998 to June 1999:

Department of Paediatrics, Civil Hospital Karachi.

House Officer

Job responsibilities included:

- **Emergency care**
- **In-patient and outpatient care**
- Assignments in the Nutrition Rehabilitation Unit and Diarrhoea Treatment Unit

OTHER PROFESSIONAL AND RESEARCH ACTIVITIES:

- **Visiting Faculty in Public Health:** Jinnah Medical College, Karachi.
- **Facilitator:** workshops in Leadership Training in Reproductive Health -organized by Population Council- November 2004 and May 2005. The participants included mid-level public health professionals from governmental and non-governmental organizations.
- **Served as Reproductive Health Specialist**: On a consultancy project evaluating fixed, outreach and community based distribution models for reproductive health services in District Khairpur (Client: Marie Stopes Society, Pakistan).
- **Editorial Assistant, National Committee for Maternal Health -now National Commission on Maternal and Neonatal Health-** (October 2003 to August 2005)
    - Preparing advocacy materials on themes relevant to Reproductive Health with particular reference to Maternal Health
- **Graduate Research Assistant**, Women's and Children's Health Policy Center, Johns Hopkins University . November 2002- May 2003
  - Conducted literature reviews on shifts in US policy and resultant changes in US domestic Maternal and Child Health legislation
  - Pilot tested an algorithm for tracking residents enrolled in community pediatrics residency programs
- **Editorial Coordinator**, Johns Hopkins Center for Communication Programs (JHUCCP). January- May 2003
  - Conducted a weekly review of research journals and news sites for articles/items on reproductive health.
  - Collated them for the JHUCCP's weekly publication the Pop Reporter.
- Supervised a survey on Reproductive Health in squatter settlements in four districts of Karachi, November 2001

- Participated in a World Health Organization and Expanded Programme of Immunization, Sindh sponsored survey of "*Qualitative Assessment of the 2000 Polio Immunization Campaign In Karachi*"
- Took primary responsibility for establishing a home school for girls in the squatter settlement that serves as Ziauddin University's primary field site.

HONOURS :

- 2009–2010 Fellow: PRB Population Policy Communications Program
- Hopkins' Sommer Scholar. A scholarship program geared towards recruiting and training "the next generation of public health leaders", it is the highest honor that the Bloomberg School of Public Health can bestow on a student
- Received the International Association of Maternal and Neonatal Health (IAMANEH) Fellowship to attend GFMER's course in Geneva.
- Recipient of the GHETS (Gender Health and Equity through Service) fellowship award and grant for incorporation of gender into the undergraduate medical curriculum.
- Received a scholarship from Johns Hopkins' Bloomberg School of Public Health for MPH.
- Recipient of the 2002 Aga Khan Foundation Scholarship (for studies at Johns Hopkins University)
- Nominated for the Delta Omega Honor Society 2002-03.

SPECIAL SKILLS:

Languages: Fluent in Urdu and English.

Computers: Proficient in Windows MS Office and Stata.

PUBLICATIONS/PRESENTATIONS

- **Khan S,** Strobino D. Maternal health, what matters: a study of individual and contextual factors related to maternal health care utilization. Paper presented at Population Association of America's 2010 annual meeting
- Gillespie D, Karklins S, Creanga A, **Khan S,** Cho N. Scaling Up Health Technologies. Report to The Bill and Melinda Gates Foundation. March 2007.
- Mujib SA, Kazmi T, **Khan S**, Shad MA, Bashir M, Khan B. Relationship of non-organic factors with malnutrition among children under three years of age. *Journal of the College of Physicians and Surgeons, Pakistan*. May 2006.
- **Khan S.** Abortion: a major contributor to maternal ill health. *Journal of the Pakistan Medical Association*. July 2005.
- Shaikh I, Omair A, **Khan S,** Inam SB, Kazmi T. Role of primary healthcare and innovative strategies in improving knowledge and practice of mothers regarding diarrhoeal diseases in a squatter settlement of Karachi. *Journal of the College of Physicians and Surgeons Pakistan*. Feb 2005.
- **Khan S,** Omair A. The impact of student intervention on Reproductive Health and Safe Motherhood Indicators Amongst Women.in a Squatter Settlement in Karachi.

Presented at Population Association of Pakistan's Fifth Annual Meeting. December 2004

- Huda N, **Khan S** " The Female Community Health Worker Training Programme: A Four-Year Experience". Presented at The Network: Towards Unity for Health 's annual conference at Atlanta, Georgia. October 2004
- Kazmi T, **Khan S** "Nutritional Status of Children under 5 in Four Districts of Pakistan". Paper presented at the annual seminar of the Pakistan Society of Physicians, April 2004.
- Inam SN, **Khan S,** "Importance Of Antenatal Care In Reduction Of Maternal Morbidity And Mortality" *Journal of the Pakistan Medical Association*. April 2002
- **Khan S**, Anjum Q, " Risk Factors for Infant Mortality in a Squatter Settlement in Karachi" Poster Presentation at the Pakistan Society of Physicians, June 2001.

Submitted for Publication:
Bharmal F, **Khan S,** Omair A. Estimating The Prevalence Of Malnutrition In Children Under Five In An Urban Squatter Settlement Using Mid-Arm Circumference As a Screening Tool.

Papers in Preparation:
Khan S and Strobino D. Delivery Care in Pakistan: A Contextual Analysis
Khan S and Strobino D. Postpartum Care in Pakistan: Factors Related to Care Seeking and Choice of Provider
Khan S and Strobino D. Maternal Mortality in Pakistan: Causes and Correlates

CURRICUL VITAE OF RESEARCHER – THE FORISCA STUDY

CHHOUN CHAMNAN, MSc, PHD

Nationality: Cambodian **Born**: 1969
Affiliation: Department of Post-Harvest Technologies and Quality Control, Fisheries Administration, No.186 Norodom Blvd., Phnom Penh, Cambodia

---

Degrees:
1992 B.Sc. in Fisheries Science, Royal University of Agriculture, Cambodia,
2001 M.Sc. in Molecular Cell Biology, Ehime University, Japan
2004 Ph.D. in Molecular Cell Biology, Ehime University, Japan

Employment
2004-08 Deputy Director of Inland Fisheries Research and Development Institute (IFReDI)
2008- Director of the Department of Fisheries Post-Harvest Technologies and Quality Control, Fisheries Administration, Ministry of Agriculture, Forestry and Fisheries.
2008- Project Investigator (PI) of WinFood in Cambodia
2012 - Project Investigator (PI) of FORISCA in Cambodia

Main research interests:
Main research area have been undertaken within food safety, food security and nutrition in developing countries, socio-economics, and toxicological research. Most research interest is in human nutrition and food safety.

Selected peer-reviewed publications:

1. **Chamnan Ch**., et al., 2009. The Role of Fisheries Resources in Rural Cambodia: Combating Micronutrient Deficiencies in Women and Children. DFPTQ/FiA. Cambodia 106p
2. Nanna R., **Chamnan C**., Deap L., Jette J., and Shakunthala H.T. (2006). Freshwater fish as a dietary source of vitamin A in Cambodia. Journal of Food Chemistry, Elservier. *In press*
3. Nanna R., Wahab M. A., **Chamnan C**., and Shakunthala H.T. (2006). Understanding the links between agriculture and health. Fish and Health. Brief 10. IFPRI, Washington, D.C. 20006-1002.USA
4. **Chamnan, C**., (2004). Molecular Cloning and Expression Analyses of the Genes Coding for Makorin ring finger protein 1 and 3-Methycrotonyl CoA Carboxylase and Pyruvate Carboxylase in Marine Fish. The dissertation accepted and published by the United Graduate School of Agricultural Sciences, Japan.

5. Abe, S., **Chamnan, C**., Miyamoto, K., Minamino, Y., and Nouda, M., (2004). Isolation and identification of 3-methylcrotonyl CoAcarboxylase and pyruvate carboxylase, and their expression in red sea bream, Pagrus major organs. Marine Biotechnology, 6 (6):527 – 540.
6. **Chamnan, C**., Abe, S., Doi, M., Chiba, S., and Gray, T.A., (2003). The genomic organization of MKRN, and expression profiles of MKRN1, MKRN2, and RAF1 in yellowtail fish*, Seriola quinqueradiata*. J. Egypt. Ger. Soc. Zool, 42C: 57-75

ANNEX 9 --- EARLIER RESEARCH ON SAFETY AND EFFICACY OF FORTIFIED RICE

(see http://www.path.org/publications/files/MCHN_u_r_res_sum_tbl.pdf)

PATH

Ultra Rice Research Summary Table

April 22, 2011

Study Code Number	Type of Research	Research Organization	Country/ Year	Study Objectives	Study Methods	Study Results	Population Studied/ Sample Size	Study Details	Publication Status	Reference
1	Safety	PATH Canada, Ottawa, Canada	Brazil, Canada 1999	To assess the optimal fortification level in Ultra Rice (UR) grains for alleviating vitamin A deficiency in young children, and to determine the upper limit of vitamin A based on the potential for toxicity to developing fetuses.	Theoretical assessment.	The maximum level of vitamin A required to meet normative requirements in children is 17 IU/g of cooked rice. This is well under the upper limit (30 IU/g) considered safe for consumption by pregnant women.	No clinical research conducted.	*Formulation studied:* Vitamin A (retinyl palmitate); *Fortification level used:* 1500 IU vitamin A per gram Ultra Rice, blended with normal rice at a ratio of 1:200	Unpublished report prepared for PATH	Berti PR, Fitzgerald S. "Advancement of Vitamin A Fortified Ultra Rice"
2	Safety	PATH Canada, Ontario, Canada	Brazil, Canada 2001	To assess circulating retinol levels after ingestion of rice fortified with Ultra Rice grains (comparing vitamin A level at 6 and 12 times the RDA per single meal).	Participants received either 10,000 IU or 20,000 IU vitamin A per 100 g uncooked rice. Serum retinol was measured at baseline and 3, 5, 7, and 24 hours after ingestion. Participants did not fast and had no dietary restrictions.	Serum retinol increased on average 25%–50% above baseline levels, and no individual increased more than 2.5 times baseline levels. Consumption of high levels of rice fortified with Ultra Rice grains (i.e., 600 g of uncooked rice = 30,000 IU in this study) is expected to provide vitamin A well below the levels suspected to be unsafe.	26 nonpregnant women in Toronto, Canada, and 25 nonpregnant women in Recife, Brazil.	*Source of UR supply:* Bon Dente International, Inc.; *Duration of consumption:* 1 meal; *Formulation studied:* Vitamin A (retinyl palmitate); *Baseline biomarkers:* Serum retinol	Unpublished report prepared for PATH	Berti PR, Krasevec J, Flores H, Schauer C. "Total Serum Retinol Levels After Consumption of Vitamin A Fortified Ultra Rice"
3	Safety	Department of Nutrition, Center of Health Sciences, Federal University of Pernambuco, Recife, Brazil	Brazil 1994	To assess the potential for vitamin A toxicity after consumption of rice fortified with Ultra Rice grains.	Participant consumed 66,000 IU of vitamin A daily (i.e., more than 25 times the daily dose intended for the children).	No adverse effects were detected either by clinical examination or standard laboratory evaluation of serum retinol, glycemia, hemogram, blood urea, serum total protein, and serum lipids.	1 adult volunteer.	*Source of UR supply:* Bon Dente International, Inc.; *Duration of consumption:* 20 days; *Formulation studied:* Vitamin A (retinyl palmitate); *Baseline biomarkers:* Serum retinol, glycemia, hemogram, blood urea, serum total protein, serum lipids	Journal of Food Science, 1994;59(2):371-372,377	Flores H, Guerra NB, Cavalcanti ACA, Campos FACS, Azevedo MCNA, Silva MBM. "Bioavailability of Vitamin A in a synthetic rice premix"
4	Stability	Department of Food Technology, Iowa State University, Ames, Iowa, USA	United States 1989	To assess the effect of two different vitamin A fortificants and different antioxidants on the stability of Ultra Rice grains.	Analyze rinse resistance, cooking stability, and shelf life using an accelerated storage stability study.	Ultra Rice formulations prepared with retinyl palmitate 250 SD and higher antioxidant levels showed a more rinse-stable product with longer shelf life. Minimizing heat and oxygen exposure during production and storage would lengthen shelf life.	No clinical research conducted.	*Source of UR supply:* Bon Dente International, Inc.; *Duration of storage study:* 12 weeks; *Formulation studied:* Vitamin A	Unpublished study supported by U.S. Department of Agriculture, Office of International Cooperation and Development, and the U.S. Agency for International Development, Bureau for Science and Technology, Office of Nutrition	Murphy PA, Fratzke A, Hauck C, O' Connor K. "Fortification of Ultra Rice with Vitamin A"
5	Stability	Department of Food Science & Human Nutrition, Iowa State University, Ames, Iowa, USA; Minnesota Valley Testing Laboratories, New Ulm, Minnesota, USA	United States 1992	To assess the effect of different formulation ingredients on the stability of Ultra Rice grains.	Analyze rinse resistance, cooking stability, and shelf life using an accelerated storage stability study.	Ascorbate was critical to maintain or improve stability at high humidity. Combinations of more saturated oils and multiple antioxidants increased vitamin A stability.	No clinical research conducted.	*Source of UR supply:* Bon Dente International, Inc.; *Duration of storage study:* Not provided; *Formulation studied:* Vitamin A (retinyl palmitate)	Journal of Food Science, 1992;57(2):437-439	Murphy PA, Smith B, Hauck C, O' Connor K. "Stabilization of Vitamin A in a synthetic rice premix"
3	Stability	Department of Nutrition, Center of Health Sciences, Federal University of Pernambuco, Recife, Brazil	Brazil 1994	To assess the nutrient retention of Ultra Rice prepared with all-trans-retinyl-palmitate during storage and to study the influence of cooking on the vitamin A content of Ultra Rice.	Samples tested for stability were stored out of direct sunlight at about 26°C and analyzed for vitamin A at 4 intervals over a six-month period. Cooked samples were boiled ~5 min followed by 20-25 min under low heat.	A six-month stability study showed initial vitamin A losses of about 25% after which the values stabilized. The loss of vitamin A from 70 samples during normal cooking was 25.9+ 9.1%.	No clinical research conducted.	*Source of UR supply:* Bon Dente International, Inc.; *Duration of storage study:* 180 days; *Formulation studied:* Vitamin A (retinyl palmitate)	Journal of Food Science, 1994;59(2):371-372,377	Flores H, Guerra NB, Cavalcanti ACA, Campos FACS, Azevedo MCNA, Silva MBM. "Bioavailability of Vitamin A in a synthetic rice premix"

Study Code Number	Type of Research	Research Organization	Country/ Year	Study Objectives	Study Methods	Study Results	Population Studied/ Sample Size	Study Details	Publication Status	Reference
6	Stability	Department of Food Science & Human Nutrition, Iowa State University, Ames, Iowa, USA	United States 1996	To simulate the effect of tropical temperature and humidity to assess the stability of Ultra Rice grains, including formulations combining both vitamin A and iron.	Analyze rinse resistance, cooking stability, and shelf life using an accelerated storage stability study.	The type of antioxidants and lipids used in the formulation were significant factors in the stability of vitamin A. Co-fortification of vitamin A and iron was not successful due to discoloration and oxidation of vitamin A.	No clinical research conducted.	***Source of UR supply:*** Bon Dente International, Inc.; ***Duration of storage study:*** Not provided; ***Formulation studied:*** Vitamin A (retinyl palmitate) only; vitamin A and iron	Food Technology, 1996;69-74	Murphy PA. "Technology of vitamin A fortification of foods in developing countries"
7	Stability	Department of Food Science and Technology, University of Georgia, Athens, Georgia, USA; PATH, Seattle, Washington, USA	United States 2000	To test the stability of vitamin A in Ultra Rice grains during cooking and after 6 months storage.	Analyze rinse resistance, cooking stability, and shelf life using an accelerated storage stability study.	The stability of vitamin A was affected much more by temperature than variation of relative humidity.	No clinical research conducted.	***Source of UR supply:*** Bon Dente International, Inc.; ***Duration of storage study:*** 6 months; ***Formulation studied:*** Vitamin A (retinyl palmitate)	Journal of Food Science, 2000;65:915-919	Lee J, Hamer ML, Eitenmiller RR. "Stability of retinyl palmitate during cooking and storing rice fortified with Ultra Rice fortification technology"
8	Stability	Philippines National Food Authority (NFA), Manila, Philippines	Philippines 2002	To determine the retention of iron after washing and cooking iron fortified rice, and to determine the effect of Ultra Rice grains on the color and flavor of cooked fortified rice.	Analyze rinse resistance and cooking stability.	Iron retention of cooked fortified rice varied depending on the blend ratio. Fortified rice made from Ultra Rice grains had similar color and flavor and better iron retention than the iron rice-premix made by NFA.	No clinical research conducted.	***Source of UR supply:*** Bon Dente International, Inc.; ***Duration of storage study:*** No storage study conducted; ***Formulation studied:*** Iron (ferrous sulfate)	Unpublished report	Philippines National Food Authority (NFA), Food Development Center. "Iron Retention of Ultra Rice Premix in the Iron Fortified Cooked Rice." Manila, Philippines: NFA; 2002
9	Stability	Southern Regional Research Center, Agricultural Research Service, United States Department of Agriculture; New Orleans, Louisiana, USA	United States 2000	To determine how differing sources and amounts of iron fortificant affect the oxidation properties of rice.	Product stability was determined by gas chromatographic analysis of lipid oxidation products. The storage variables included temperature and packaging.	Rice fortified with elemental iron alone or with multiple fortificants had better storage characteristics than that fortified with ferrous sulfate alone.	No clinical research conducted.	***Source of UR supply:*** Bon Dente International, Inc.; ***Duration of storage study:*** 6 months; ***Formulations studied:*** Iron (ferrous sulfate or elemental iron), thiamin (thiamin mononitrate), folic acid and zinc (zinc oxide)	Cereal Chemistry. 2004;81:384-388. Based on research done in 2000.	Bett-Garber KL, Champagne ET, Ingram DA, Grimm CC. "Impact of iron source and concentration on rice flavor using a simulated rice kernel micronutrient delivery system"
10	Stability	Food BioTek Corp., Toronto, Canada	Canada 2003	To review, test, and reformulate the antioxidant components of the Ultra Rice formula to improve stability and meet international food standards.	Twenty formulations were tested, including 4 different vitamin A powders and 10 different antioxidants. Stability was analyzed under varying temperature and relative humidity conditions.	The best formulations retained nearly 80% of added vitamin A even after 24 weeks at 45°C and 60% humidity. This was better and cheaper than the original Ultra Rice formulation and included only Codex Alimentarius-compliant ingredients.	No clinical research conducted.	***Source of UR supply:*** University of Toronto; ***Duration of storage study:*** 6 months; ***Formulation studied:*** Vitamin A (retinyl palmitate)	Unpublished report prepared for PATH	"Antioxidant Systems for the Preservation of Vitamin A in Ultra Rice"
11	Stability	Medallion Laboratories, Minneapolis, Minnesota, USA	Colombia, United States 2004	A storage study was conducted to determine the stability of the original vitamin A premix over time and to assess whether the micronutrient overages used for production were adequate.	Samples from a single production lot were separated into two different batches—one stored frozen and the second stored at ambient conditions. Over a period of 9 months, nutrient levels from both the frozen and ambient samples were analyzed and compared.	Micronutrient levels varied depending on whether the samples were stored under frozen or ambient conditions. Zinc is stable regardless of storage conditions. Vitamin E is stable for 9 months if the premix is frozen, but begins to oxidize after about 4 months under ambient conditions. Losses of folic acid were comparable under both storage conditions. Vitamin A was very unstable, with only 30% remaining after one month storage under ambient conditions. Thiamin results were inconclusive.	No clinical research conducted.	***Source of UR supply:*** Union de Arroceros, S.A.; ***Duration of storage study:*** 9 months; ***Formulation studied:*** Vitamin A (retinyl palmitate), vitamin E (d-l alpha tocopherol), thiamin (thiamin mononitrate), folic acid, and zinc (zinc oxide)	Unpublished report prepared for PATH.	Rief D. "Comparative Stability Study Using Colombian Fortified Premix"
12	Stability	Food BioTek Corp., Toronto, Canada	Canada 2005	To expand on earlier research to develop an Ultra Rice formulation incorporating iron, zinc, and multiple B vitamins that maintains its color and iron bioavailability, as well as most of its vitamin B activity, over the normal shelf life of Ultra Rice.	Sixteen different formulations were made using 4 different iron compounds and 4 different antioxidant combinations. Stability studies were conducted at varying temperature and relative humidity conditions. Samples were examined each 6 to 8 weeks and analyzed for micronutrient content, color, and rancidity.	It is feasible to produce an Ultra Rice premix with a good shelf life combining several B vitamins, iron, and zinc with good micronutrient stability. The current Ultra Rice formulation possesses good micronutrient stability and undergoes minimal changes in color and flavor, even under harsh environmental conditions, for periods up to 6 months.	No clinical research conducted.	***Source of UR supply:*** University of Toronto; ***Duration of storage study:*** 6 months; ***Formulation studied:*** Iron (ferric pyrophosphate, ferrous fumarate), thiamin (thiamin mononitrate), folic acid, niacin (niacinamide) and zinc (zinc oxide)	Li Y, Diosady LL, Jankowski S. 2006. Effect of iron compounds on the storage stability of multiple-fortified Ultra Rice. International Journal of Food Science and Technology, Online Early Articles. Published article online: June 21, 2007.	Diosady L, Li OY. "Final report on Phase II report on the development and stability testing of an iron-containing formulation of Ultra Rice"

Study Code Number	Type of Research	Research Organization	Country/ Year	Study Objectives	Study Methods	Study Results	Population Studied/ Sample Size	Study Details	Publication Status	Reference
13	Stability	Food BioTek Corp., Toronto, Canada	Canada 2006	To investigate the stability of thiamin (vitamin B1) and its effects on organoleptic properties in Ultra Rice in the presence of encapsulated ferrous fumarate.	Three formulas were produced containing ferrous fumarate, thiamin, and three different antioxidant combinations. Stability studies were conducted at varying temperature and relative humidity conditions with measurements at 6,12, and 20 weeks.	This study demonstrated the feasibility of incorporating encapsulated ferrous fumarate in a stable Ultra Rice formulation containing vitamin B1. When incorporated into simulated rice grains, its bioavailability and stability were not affected.	No clinical research conducted.	***Source of UR supply:*** University of Toronto; **Duration of storage:** 20 weeks; ***Formulation studied:*** Iron (ferrous fumarate), thiamin (thiamin mononitrate)	International Journal of Food Sciences and Nutrition. 2008;59(1):24-33	Li Y, Diosady L, Jankowski S. "Stability of vitamin B1 in Ultra Rice in the presence of encapsulated ferrous fumarate"
3	Bioavailability	Department of Nutrition, Center of Health Sciences, Federal University of Pernambuco, Recife, Brazil	Brazil 1994	To assess the bioavailability of vitamin A in Ultra Rice grains using the relative dose response test.	A relative dose response test was conducted measuring pre- and post-challenge dose serum retinol values to indirectly show the status of vitamin A reserves in the liver.	The fact that deficient subjects showed a serum retinol reaction in a 5-hour period indicates that the retinol in Ultra Rice was absorbed and transported.	83 children aged 11-77 months.	***Source of UR supply:*** Bon Dente International, Inc.; ***Duration of consumption:*** 1 meal; ***Formulation studied:*** Vitamin A (retinyl palmitate); ***Baseline biomarkers:*** Serum retinol	Journal of Food Science 1994;59(2):371-372,377	Flores H, Guerra NB, Cavalcanti ACA, Campos FACS, Azevedo MCNA, Silva MBM. "Bioavailability of Vitamin A in a synthetic rice premix"
14	Bioavailability	Human Nutrition Research Center, Agricultural Research Center, United States Department of Agriculture, Grand Forks, North Dakota, USA.	United States 2003	To determine the relative bioavailability (RBV) of iron from four ferric pyrophosphate compounds using the AOAC Rat Hemoglobin Repletion Method.	Nineteen different dietary treatment groups were evaluated. The rats were weighed and their Hb levels were measured, both following a low-iron depletion diet of 24 days, and after consuming an iron-fortified diet for 14 days. Hb repletion data were analyzed by the slope ratio assay method, expressing bioavailability relative to ferrous sulfate.	SunActive® iron was more bioavailable than the other ferric pyrophosphate compounds for correcting the iron deficiency of anemic rats (92%–94% RBV). The bioavailability of the other ferric pyrophosphate compounds was approximately 70%–75% RBV.	171 weanling male Sprague-Dawley rats (approx. 83 g) were randomized into three cohorts (57 rats per cohort) and given 24 days of an iron depletion diet followed by a 14-day repletion period.	***Duration of consumption:*** 14 days after 24 day depletion period; ***Formulation studied:*** Iron (ferric pyrophosphate and ferrous sulfate); ***Baseline biomarkers:*** Blood hemoglobin (Hb)	Unpublished report prepared for PATH.	Hunt JR. "Assessment of the Bioavailability of Ferric Pyrophosphate in Ultra Rice Using the AOAC Rat Hemoglobin Repletion Method"
15	Efficacy	Department of Nutrition, Center of Health Sciences, Federal University of Pernambuco, Recife, Brazil	Brazil 1994	To assess the biological efficacy of vitamin A fortified Ultra Rice grains.	Participants received vitamin A fortified rice during a 1-month period. Data on serum retinol level, anthropometry, clinical status of vitamin A deficiency, childhood co-morbidity, and dietary intakes were evaluated before and after the study period.	Following the feeding period, 8.8% of the children presented with serum retinol levels below the proposed cut-off point of 1.05 umol/L compared to 51% of the children at baseline, showing that consuming fortified rice improved serum retinol status. Although all children did not reach satisfactory vitamin A status during the feeding period, the frequency distribution of serum retinol values showed a significant shift to the right. No significant differences between control and experimental groups were observed in morbidity or anthropometric parameters.	83 children aged 11-77 months.	***Source of UR supply:*** Bon Dente International, Inc.; ***Duration of consumption:*** 30 days; ***Formulation studied:*** Vitamin A (retinyl palmitate); ***Baseline biomarkers:*** Serum retinol	Unpublished report.	Flores H, Campos FACS, Silva MBM, Barretto E, Albuquerque S. "Efficacy of Vitamin A Enriched Rice in the Treatment and Prevention of Vitamin A Deficiency." Recife, Brazil
16	Efficacy	Program in International Nutrition, Department of Nutrition, University of California-Davis, USA; Nepali Technical Assistance Group, Kathmandu, Nepal	Nepal, United States	To assess the effect of daily consumption of small doses of vitamin A from various natural food sources, vitamin A-fortified rice, and retinyl palmitate supplement for treating vitamin A deficiency and improving plasma retinol concentrations in night-blind pregnant Nepali women.	Night-blind women were randomly assigned to 1 of 6 treatment groups to receive 6 d/wk for 6 wks either 850 mcg REd as retinyl palmitate, vitamin A fortified rice, goat liver, GLV, carrots or 2000 mcg REd as a capsule. Plasma concentrations of retinol, carotenoids, tocopherols, ferritin, zinc, Hb, and C-reactive protein were measured as well as anthropometry taken before and after the intervention period. At weekly visits, frequency of consuming vitamin A rich foods, and dark adaptation was assessed by pupillary response threshold.	In night-blind women the mean pupillary response threshold improved significantly in response to intervention. Improvement in dark adaptation was greatest in the group consuming liver. Plasma retinol concentration increased significantly in the higher-dose capsule and liver groups as compared to other groups, and was significantly greater in the liver group than in vitamin A fortified rice. Improvement in dark adaptation did not differ significantly between women who received vitamin A as liver, GLV, carrots, capsule, or fortified Ultra Rice.	348 pregnant nightblind Nepali women.	***Source of UR supply:*** Bon Dente International, Inc.; ***Duration of consumption:*** 6 weeks; ***Formulation studied:*** Vitamin A; ***Baseline biomarkers*** : Pupillary response, plasma retinol	American Journal of Clinical Nutrition, 2005;81:461-1. Study initiated in 2000; completed in 2003.	Haskell MJ, Pandey P, Graham JM, Shrestha RK, Brown KH. "Recovery from impaired dark adaptation in night-blind pregnant Nepali women who receive small daily doses of vitamin A as amaranth leaves, carrots, goat liver, vitamin A-fortified rice or retinyl palmitate"
17	Efficacy	Centro de Investigacion en Nutricion y Salud; PATH	Mexico 2005	To test the efficacy of rice fortified with microencapsulated, micronized pyrophosphate (Sun Active Fe) to improve the iron status of women in Mexico consuming 20 mg iron per day.	In a randomized, blinded, placebo-controlled feeding trial, women were recruited from 6 factories and received a daily portion of cooked rice 5 days per week for a period of 6 months. At baseline and endline, venous blood was collected for biochemical analysis. At midline, capillary blood obtained by the finger prick method was analyzed for Hb and urine for pregnancy.	In the group eating fortified rice, anemia was reduced by 80% and iron deficiency was reduced by 29%. Mean plasma ferritin concentration and estimated body iron stores were significantly higher, and transferrin receptors were lower in the iron-fortified group.	180 nonpregnant non-lactating women aged 18-49 years.	***Source of UR supply:*** *University of Toronto;* ***Duration of consumption:*** 6 *months* ***Formula studied:*** *Iron (ferric pyrophosphate)* ***Baseline biomarkers:*** *Plasma ferritin, transferrin receptor, C-reactive protein*	Food and Nutrition Bulletin. 2008; 29(2):140-149.	Hotz C, Porcayo M, Onofre G, Garcia-Guerra A, Elliott T, Jankowski S, Greiner T. "Efficacy of iron-fortified Ultra Rice in improving the iron status of women in Mexico"

Study Code Number	Type of Research	Research Organization	Country/ Year	Study Objectives	Study Methods	Study Results	Population Studied/ Sample Size	Study Details	Publication Status	Reference
15	Effectiveness	Department of Nutrition, Center of Health Sciences, Federal University of Pernambuco, Recife, Brazil	Brazil 1994	To assess the effectiveness of including vitamin A- fortified rice in the normal food given to preschool children.	Fasting blood samples were taken at baseline, and then at 4-month intervals, to determine serum retinol. At the baseline and 12-month evaluations, the children underwent a Relative Dose Response test. Anthropometry, clinical vitamin A status, morbidity, and dietary evaluation were assessed.	Serum retinol values continually increased at each of the four monthly measurements up to 1 year of age. After a full year, no children were deficient compared to 44% at baseline.	415 children aged 7.7-83.6 months, from 6 randomly selected municipal day care centers.	***Source of UR supply:*** Bon Dente International, Inc.; ***Duration of consumption:*** 12 months; ***Formulation studied:*** Vitamin A (retinyl palmitate); ***Baseline biomarkers:*** Serum retinol	Unpublished report.	Flores H, Campos FACs, Silva MBM, Lins MH, Barretto E, Albuquerque S. "Efficacy of Vitamin A Enriched Rice in the Treatment and Prevention of Vitamin A Deficiency"
18	Effectiveness	National Institute of Nutrition, Indian Council of Medical Research, Hyderabad, India	India Aug 2007-Apr 2008	To assess the impact of consuming rice fortified with iron Ultra Rice on the iron status of children in a mid-day meal program in India.	A randomized, placebo-controlled, doubleblind feeding trial providing 18 mg iron per 125 g serving of fortified rice (group B) in comparison to consuming unfortified rice (group A). A total of 140 children were randomly grouped into group A and group B. Iron-Ultra Rice containing 9.6 mg Fe/g was blended with natural rice using a 1.6:98.4 blend ratio. Participants were assessed (height, weight, nutrition, clinical tests) at baseline and at the end of 9 months.	After intervention, there was a significant (p<0.001) increase in the mean serum ferritin levels in the experimental group (8.17Hg/dL) while a marginal decline was observed in the control group (-3.04Hg/dL). There was a significant increase in the mean haemoglobin (Hb) levels, in both the study groups. The mean CRP levels between groups were similar both at baseline and end line. There was a significant reduction in the incidence of morbidities among children in the experimental group. Conclusions: Ultra Rice improved iron stores and reduced morbidities in experimental group. Hb levels improved in both groups but was not significant between control and experimental group.	140 children aged 5-11 years with hemoglobin levels >7 g/dl and regularly participating in a mid-day meal program.	***Source of UR supply:*** Camil Alimentos, S.A.; ***Duration of consumption:*** 166 days; ***Formulation studied:*** Iron (ferric pyrophosphate, 3 um); ***Baseline biomarkers:*** Hemoglobin, serum ferritin, C-reactive protein	Unpublished report. Project funded by DBT, Govt. of India (No. BT/PR-7003/PID/20/269/2005).	Brahman GNV, et al. "Evaluation of Bio-effect of Ultra Rice on Iron Status of Beneficiaries of MDM - a Study in a School of Ranga Reddy District of Andhra Pradesh"
19	Effectiveness	Department of Pediatrics, School of Medicine, Federal University of Minas Gerais, Minas Gerais, Belo Horizonte, Brazil	Brazil 2007	To compare the effectiveness of iron-fortified rice with iron drops in improving young child iron status among families in a Southeast region of Brazil.	A randomized, double-blinded effectiveness trial comparing biochemical indicators after consuming iron-fortified Ultra Rice 5 days per week or receiving iron drops 3 times per week. Subjects were evaluated at baseline and after a 5-month period.	There was a significant improvement in iron status in both groups; however, the shift from severe deficiency to moderate/low deficiency of hemoglobin levels was significantly greater in the group receiving iron fortified rice. The findings suggest that providing rice fortified with iron Ultra Rice is an effective strategy for improving iron status in children.	175 children aged 6-24 months with a hemoglobin concentration between 8.0 and 11.0 g/dl	***Source of UR supply:*** Camil Alimentos, S.A.; ***Duration of consumption:*** 5 months; ***Formulation studied:*** Iron (ferric pyrophosphate, 3 um); ***Baseline biomarkers:*** Hemoglobin	Journal of Nutrition, 2009; 140;49–53.	Beinner MA, et al. "Iron-Fortified Rice Is As Efficacious As Supplemental Iron Drops in Infants and Young Children"
3	Sensory evaluation	Department of Nutrition, Center of Health Sciences, Federal University of Pernambuco, Recife, Brazil	Brazil 1994	To test the acceptability of rice fortified with Ultra Rice grains.	Panelists responded to "difference" and "preference" questionnaires comparing normal rice and rice fortified with Ultra Rice grains using the Lamond method (1970).	"Difference" testing showed no significant difference between the taste of normal and enriched rice. The sensory properties of enriched rice were rated higher for enriched rice based on "preference" testing.	14 panelists participated in the sensory evaluation.	***Source of UR supply:*** Bon Dente International, Inc.; ***Formulation studied:*** Vitamin A (retinyl palmitate)	Journal of Food Science, 1994;59(2):371-372,377.	Flores H, Guerra NB, Cavalcanti ACA, Campos FACS, Azevedo MCNA, Silva MBM. "Bioavailability of vitamin A in a synthetic rice premix"
9	Sensory evaluation	Southern Regional Research Center, Agricultural Research Service, United States Department of Agriculture, New Orleans, Louisiana, USA	United States 2000	To determine how differing sources of iron impact the effect of Ultra Rice on the flavor of milled rice, as determined by descriptive analysis.	Flavor was determined by smelling and evaluation in the mouth by trained panelists to assess 12 unique flavor attributes.	The effects of iron fortification on flavor were dependent on iron source and concentration.	12 panelists participated in the sensory evaluation.	***Source of UR supply:*** Bon Dente International, Inc.; ***Formulation studied:*** Iron (ferrous sulfate, elemental iron) only and iron, thiamin (thiamin mononitrate), folic acid, and zinc (zinc oxide)	Cereal Chemistry. 2004;81:384-388. Based on research done in 2000.	Bett Garber KL, Champagne ET, Ingram DA, Grimm CC. "Impact on iron source and concentration on rice flavor using a simulated rice kernel micronutrient delivery system"
20	Sensory evaluation	Indian Market Research Bureau International (IMRB), New Delhi, India	India 2003	To gauge the organoleptic acceptance of Ultra Rice fortified with ferrous sulfate and the sensitivity of potential target segments.	6 different samples of fortified rice were presented to consumers in a "blind," rotating order. Consumers ranked each sample on a five-point scale on smell, taste, aftertaste, similarity to normal rice, and overall liking.	Consumers were generally unable to sense any difference between fortified rice and regular rice and liked them equally well, although small percentages did not like the grayish color or reported an aftertaste.	600 consumers were assessed for the evaluation.	***Source of UR supply:*** Bon Dente International, Inc.; ***Formulation studied:*** Iron (ferrous sulfate), zinc (zinc oxide), thiamin (thiamin mononitrate), and folic acid	Unpublished report prepared for PATH.	IMRB. "Potential Introduction of Ultra Rice in India: Complete Market Assessment"
21	Sensory evaluation	Consultor Apoyo, Quito, Ecuador	Ecuador 2003	To evaluate consumer acceptance and preference of regular rice and rice fortified with either sodium iron EDTA or ferric pyrophosphate using Ultra Rice grains.	Consumers assessed both raw and cooked rice to evaluate appearance, taste, and aroma, and to evaluate the general concept of fortified rice and intent to purchase.	In general, regular rice was preferred over fortified rice, and samples containing ferric pyrophosphate were favored over those fortified with sodium iron EDTA. The concept of fortified rice was considered to be important among those surveyed, and respondents said they would buy it if available (4.3 on a 5-point "intent to purchase" scale).	419 participants were assessed for the evaluation.	***Source of UR supply:*** Bon Dente International, Inc.; ***Formulation studied:*** Iron (ferric pyrophosphate or sodium iron EDTA)	Unpublished report prepared for PATH.	Consultor Apoyo. "Ultra Rice Organoleptic Tests in Ecuador"

Study Code Number	Type of Research	Research Organization	Country/ Year	Study Objectives	Study Methods	Study Results	Population Studied/ Sample Size	Study Details	Publication Status	Reference
**18**	Sensory evaluation	National Institute of Nutrition, Indian Council of Medical Research, Hyderabad, India	India 2006	To test the organoleptic properties of rice fortified with iron Ultra Rice.	Participants assessed color, appearance, texture, smell, taste, and overall acceptability of cooked iron-fortified or unfortified rice using a 5-point hedonic scale. The children were randomly assigned to 2 groups and received either fortified or non-fortified rice as part of the mid-day meal program.	The overall acceptability of cooked fortified rice was 88% vs. 97% for unfortified rice. The difference in scores was not statistically significant, suggesting that both types of rice were well accepted by the children. (Note: The NIN study was initiated in 2006 and this acceptability component was completed in the initial trial. The effectiveness componenet was done again in 2007-08, the results of which are outlined above.)	134 children aged 8-11 years old participating in an Indian mid-day meal program	***Source of UR supply:*** Camil Alimentos, S.A.; ***Formulation studied:*** Iron (ferric pyrophosphate, 3 um)	Unpublished report. Project funded by DBT, Govt. of India (No. BT/PR-7003/PID/20/259/2005).	Brahman GNV, Nair KM. "Evaluation of Bio-effect of Ultra Rice on Iron Status of Beneficiaries of MDM - a Study in a School of Rangareddy District of Andhra Pradesh"
**19**	Sensory evaluation	Department of Pediatrics, School of Medicine, Federal University of Minas Gerais, Minas Gerais, Belo Horizonte, Brazil	Brazil 2007	To examine sensory differences between conventional rice and rice fortified with iron Ultra Rice and determine consumer acceptance.	Differences between both types of rice were analyzed using the Duo-Trio Test. The acceptance test evaluated general rice appearance, color, aroma, and taste using a 7-point hedonic scale with extremes ranging from really disliked to really liked.	There were no significant differences between the analyzed samples of conventional rice and iron Ultra Rice-fortified rice. The iron did not alter the rice sensory characteristics and the fortified rice was well accepted.	37 non-trained subjects assessed the difference between samples; 43 subjects analyzed the fortified rice for acceptance.	***Source of UR supply:*** Camil Alimentos, S.A.; ***Formulation studied:*** Iron (ferric pyrophosphate, 3 um)	Journal of Nutrition, 2009; 140;49–53.	Beinner MA, et al. "Iron-Fortified Rice Is As Efficacious As Supplemental Iron Drops in Infants and Young Children"
**22**	Sensory evaluation	Institut de Recherche pour le Developpement (IRD), Marseille, France	Cambodia 2010	To assess acceptability of fortified rice among mothers, teachers, directors, and students.	?	62% of teachers and 85% of mothers correctly identified the cooked fortified rice (both P<0.001). Normal and fortified rice were scored similarly for color, smell, appearance, stickiness, or hardness by teachers and partners (P>0.05). School children scored fortified rice slightly better than normal rice for taste and smell.	2,000 school children.	***Source of UR supply:*** Camil Alimentos, S.A.; ***Duration of consumption:*** XX days ***Formulation studied:*** Iron (ferric pyrophosphate, 3 um); zinc, thiamine, folic acid	Unpublished data.	Wieringa F. "UM-204 Prevention of Malnutrition." Presentation at the National Institute of Nutrition in Hanoi. IRD; 2010.
**23**	Sensory evaluation	National Institute of Nutrition (NIN), Hanoi, Vietnam	Vietnam 2010	To examine the factors affecting the intention/decision to buy fortified rice.	Cross-sectional study in 2 communes in 2 districts. **Sensory test description:** Each test taker received 3 different bowls of rice, of which 2 bowls were similar (also called repeated sample products). These 2 bowls contained either normal rice or fortified rice. The 3rd bowl contained either fortified rice or normal rice respectively. A bowl starting with an odd digit was fortified rice and a bowl starting with an even digit was normal rice. **Predilection test:** 135 subjects tried fortified rice and were then asked to rank it on a scale from "extremely disliked" to "extremely liked."	There is a significantly different sensory between fortified rice and normal rice after cooking. There is no difference between Ultra Rice and NutriRice. A predilection test showed the attitude of women toward fortified rice is quite good (negative 7.8%, neutral 34.3%, positive 60.84%). Women's knowledge of iron, vitamin A, folic acid, and micronutrients is very poor although information about these micronutrients was provided in many intervention programs. Women are willing to buy the fortified rice if they are convinced of its health benefits.	**Sensory test:** 60 subjects (triangle test, single blind test). **Predilection test:** 135 subjects. **Focus group discussion:** 4 groups of women (10 each) and 2 groups of millers (10 each).	***Source of UR supply:*** Camil Alimentos, S.A.; ***Duration of consumption:*** XX days ***Formulation studied:*** Iron (ferric pyrophosphate, 3 um); zinc, thiamine, folic acid	Unpublished data.	Tran KV. "Acceptability of Fortified Rice in Vietnamese." Presentation. NIN.
**24**	Sensory evaluation	Nicaraguan Ministry of Health, Managua, Nicaragua; United Nations Children's Fund, New York, USA	Nicarauga 2010	To establish the acceptability of fortified rice.	Each subject was given fortified rice (2 samples) and non-fortified rice (1 sample). Subjects used a ballot with a 7-point hedonic scale. The results were analyzed using a randomized block arrangement through an analysis of variance for each of the attributes evaluated (color, odor, texture, flavor, and overall liking) to establish a test performed at a level of significance of 0.05.	Ultra Rice obtained general satisfaction scores above 4 points. It was concluded that consumers perceive positive sensory attributes and acceptability of rice fortified with Ultra Rice.	50 people from different social strata. The assessment took place in the dining facilities of the Autonomous University of Nicaragua (UNAN) in cubicle-like spaces to minimize interference emission of comments from participants.	***Source of UR supply:*** Adorella Alimentos, Brazil; ***Formulation studied:*** Iron (ferric pyrophosphate, 3 um); zinc, thiamine, folic acid	Published UNICEF report.	Reyes CM, Alvarado M. "Technology assessment of rice fortification in Nicaragua"
**25**	Nutrient retention	PATH; Dourados Municipality, Brazil; Embrapa, Brasilia, Brazil	Brazil 2010	To monitor both nutrient retention and compliance of kitchen staff implementing the school meal pilot in Dourados.	Kitchen cooks received training from PATH on the proper blending procedure prior to the start of the pilot. Samples were collected at 3 of the 7 pilot schools. Each 400g sample was analyzed for iron and zinc content by Embrapa laboratories in Dourados--samples were ashed, and the resulting ash was further homogenized before taking sub-samples for measurement of the micronutrient content.	Coefficient of variation between expected and observed levels of Fe and Zn were calculated. The baseline content of Fe and Zn in the Ultra Rice was analyzed by the laboratory at Embrapa--this enabled the team to calculate the expected value for each mineral in the cooked fortified rice samples. The difference between mean observed value and expected values in each school were as follows: School #1 - Fe 14%, Zn 25%; School #2 - Fe 21%, Zn 10%; School #3 - Fe 11%, Zn 26%. Sample-to-sample variation expressed by CVs were all within an acceptable range, suggesting: 1) The chefs complied with the blending protocol and added the proper amount of Ultra Rice per batch; and 2) micronutrients were retained from baseline to post cooking.	400g samples (total, n=12) of cooked fortified rice were taken from the top (n=3), middle (n=3), and bottom (n=3) of a single cooking vessel in each of the 3 schools.	***Source of UR supply:*** Adorella Alimentos, Brazil; ***Formulation studied:*** Iron (ferric pyrophosphate, 3 um); zinc, thiamine, folic acid	Unpublished data: Lab analysis commissioned by PATH. Conducted at third-party laboratory, Silliker Labs Canada, Markham Ontario.	

Study Code Number	Type of Research	Research Organization	Country/ Year	Study Objectives	Study Methods	Study Results	Population Studied/ Sample Size	Study Details	Publication Status	Reference
26	Nutrient retention	PATH; Naandi Foundation, Hyderabad, India; Global Alliance for Improved Nutrition (GAIN), Geneva, Switzerland	India 2009	To monitor nutrient retention of fortified rice cooked by 2 different methods and by 2 different preparation methods; also to monitor the compliance of kitchen staff in adding the proper amount of Ultra Rice.	***Cooking methods*** : Rice is cooked by 2 methods: absorption in which all water is absorbed, and excess water in which water not absorbed during cooked is discarded. Six 100g samples of cooked rice from both methods were collected. ***Preparation methods*** : In addition, nutrient retention after the 2 preparation techniques was evaluated: 1) Ultra Rice was added directly to the cooking vessel and 2) Ultra Rice was added to soaking tubs before rice was transferred to the larger rice cookers. The latter method was preferred because kitchen workers had difficulty properly mixing the Ultra Rice grains in the extremely large 125kg cauldrons of rice. This analysis helped determine whether exposing the Ultra Rice grains to a pre-soaking procedure would reduce the iron content in the cooked rice (due to a potential breach of integrity from greater exposure to water).	***Cooking methods*** : Mean Fe content of samples cooked by excess water (values in mg/100 grams): 14.1 + 8.33; by absorption method: 19.96 + 8.4. The difference between the observed and expected Fe content (20mg) was minimal when cooking by absorption. For the excess water method, however, there was approximately 30% loss of Fe. ***Preparation methods*** : The difference in iron (values in mg/100 grams) content of cooked rice when Ultra Rice was added directly to the cooking vessel or to the tubs with soaked rice was not statistically significant (4.24 + 2.5 when adding directly to the cooking vessel; 6.7 + 4.9 when adding to soaking tubs). These samples were cooked in excess water and hence their iron content was lower than what would be found through the absorption method of cooking. Note: the difference in reported Fe content between the 2 samples that were cooked in excess water was related to the fact that 1 sample was not reported on a dry basis (i.e., no moisture content was given by the lab). Therefore, they could not be directly compared.	Six 100g samples were taken for each cooking method and preparation method.	***Source of UR supply:*** Swagat Foods, India; **Formulation studied:** Iron (ferric pyrophosphate, 3 um)	Unpublished data: prepared in final report for GAIN.	"GAIN-Naandi-PATH Pilot Study: A comprehensive report." PATH; October 2010.
27	Nutrient retention	Silliker Laboratories, Ontario, Canada	Canada 2010	To determine the nutrient retention of Ultra Rice grains after rigorous cooking and preparation practices.	Soaking, rinsing, and cooking tests were carried out on individual 100g samples of blended, fortified rice. Each 100g sample contained 99.5g traditional rice and 0.5g Ultra Rice. Iron and thiamine content were analyzed at baseline, and then after the following procedures: 1) rinsing; 2) rinsing and soaking; 3) rinsing, soaking, and cooking in excess water; 4) rinsing, soaking, and cooking by absorption; 5) rinsing, soaking, frying in oil, and then cooking by absoprtion.	Fe baseline: 13.3 +/- 1.72 (values in mg/100g). Virtually no iron was lost after rinsing, soaking, and cooking by both methods. Fe losses: frying in oil did result in 46% loss of iron from baseline (7.15+/-3.74); vitamin B1 baseline :1.14+/- 0.07 (values in mg/100g); vitamin B1 losses (values in mg/100g): 23% after rinsing (0.88); 30% after rinsing and soaking (0.80 +/- 0.28); 17.5% after rinsing, soaking, and cooking by absorption (0.94+/- 0.02); and 39% after rinsing, soaking, pan frying, and cooking by excess water (0.69). There were not enough samples to determine whether the greater losses seen in Fe compared to vitamin B1 after pan frying (46% vs. 39%) were statistically significant.	Number of 100g samples "n" taken at each step: baseline (n=3); rinsing (n=1); rinsing and soaking (n=2); cooking by absorption (n=2); cooking in excess water (n=2); frying in oil (n=3).	***Source of UR supply:*** Adorella Alimentos, Brazil; **Formulation studied:** Iron (ferric pyrophosphate, 3 um); zinc, thiamine, folic acid	Unpublished data: Lab analysis commissioned by PATH, conducted by third-party laboratory, Silliker Labs Canada, Markham Ontario.	
28	Blend homogeneity	PATH; Naandi Foundation, Hyderabad, India; Global Alliance for Improved Nutrition (GAIN), Geneva, Switzerland	India 2009	To evaluate blend homogeneity of fortified rice using 2 blending methods: point-of-use blending (Phase A) and blending further up in the supply chain at a rice mill (Phase B).	*Phase A* : 1) Inter-batch variation (same chef) - samples were taken from 3 batches of fortified rice to measure mean iron content; 2) Inter-chef variation - samples were taken from 3 batches prepared by different chefs to analyze mean iron content in each batch.<br>*Phase B* : Iron content was analyzed from samples of both dry-blended fortified rice, and after this dry-blended fortified rice was cooked, as described above.	***Phase A*** : The mean coefficient of variation in iron content was found to be 47%. Interbatch variation between 2 of the 3 batches was insignificant (Batch #1: 6.7 + 4.9, Batch #3: 6.5 + 3.6) but samples from Batch #2 were much lower than expected (2.9 + 0.9), possibly due to lack of chef compliance. Inter-chef variation: Little difference was seen in the mean iron content between 2 chefs (Chef #1: 6.7 + 4.9; Chef #2: 2.9 + 1.0; Chef #3: 3.3 + 1.0).<br>***Phase B:*** The CV of the dry, uncooked fortified rice was found to be 17% - well within the standard acceptable range for other fortified foods which is around 20-25%; after cooking, the CV increased to 39%. It is unclear as to why the homogeneity decreased after cooking. To further investigate this issue, larger samples sizes will be used in the next operational trial to rule out an artificial sampling error as a reason for the increase.<br>***Comparing Phase A and Phase B:*** The mean co-efficient of variation of iron content in cooked rice was used as a test for homogeneity, and found to be 47% in Phase A and 39% in Phase B. With a p-value <0.00001, the difference in variance or homogeneity between wet blend and dry blend is significant, with dry blend proving to be better. However, both CV values are high. This could be due to a sampling problems at the lab--the cooked fortified rice samples may not have been adequately homogenized before sub-sampling, confounding the results.	Samples of cooked rice weighing 250g each were drawn from top, middle, and bottom layers of cooked rice from the cauldron.	***Source of UR supply:*** Swagat Foods, India; **Formulation studied:** Iron (ferric pyrophosphate, 3 um)	Unpublished data: prepared in final report for GAIN.	"GAIN-Naandi-PATH Pilot Study: A comprehensive report." PATH; October 2010.
29	Blend homogeneity	PATH; Dourados Municipality, Brazil; Embrapa, Brasilia, Brazil	Brazil 2010	To evaluate blend homogeneity of fortified rice using the point-of-use method in school lunch programs.	Kitchen cooks received training from PATH on the proper blending procedure prior to the start of the pilot. Samples were collected at 3 of the 7 pilot schools. Each 400g sample was analyzed for iron and zinc content by Embrapa laboratories in Dourados--samples were ashed, and the resulting ash was further homogenized before taking the sub-samples.	The results confirmed that the cooks were properly mixing the Ultra Rice into traditional rice and the resulting mixture was at or close to the industry standard for acceptable CV for fortified foods (20%). CV of Fe content between 12 samples: School #1 - 20%; School #2 - 31%; School #3 - 21%; CV of Zn content between 12 samples: School #1 - 21%; School #2 - 11%; School #3 - 17%.	Twelve 400g samples of cooked fortified rice were taken from the top (n=3), middle (n=3), and bottom (n=3) of a single cooking vessel in each of the 3 schools.	***Source of UR supply:*** Adorella Alimentos, Brazil; **Formulation studied:** Iron (ferric pyrophosphate, 3 um); zinc, thiamine, folic acid	Unpublished data.	
